# Author Correction: Deep learning and genome-wide association meta-analyses of bone marrow adiposity in the UK Biobank

**DOI:** 10.1038/s41467-025-59574-9

**Published:** 2025-05-09

**Authors:** Wei Xu, Ines Mesa-Eguiagaray, David M. Morris, Chengjia Wang, Calum D. Gray, Samuel Sjöström, Giorgos Papanastasiou, Sammy Badr, Julien Paccou, Xue Li, Paul R. H. J. Timmers, Maria Timofeeva, Susan M. Farrington, Malcolm G. Dunlop, Scott I. Semple, Tom MacGillivray, Evropi Theodoratou, William P. Cawthorn

**Affiliations:** 1 Centre for Global Health and Molecular Epidemiology, Usher Institute, University of Edinburgh, Edinburgh, UK; 2 University/BHF Centre for Cardiovascular Science, University of Edinburgh, The Queen’s Medical Research Institute, Edinburgh BioQuarter, 47 Little France Crescent, Edinburgh, UK; 3 Edinburgh Imaging, University of Edinburgh, The Queen’s Medical Research Institute, Edinburgh BioQuarter, 47 Little France Crescent, Edinburgh, UK; 4 School of Mathematics and Computer Sciences, Heriot-Watt University, Edinburgh, UK; 5 Archimedes Unit, Athena Research Centre, Marousi, Greece; 6 Univ. Lille, CHU Lille, Marrow Adiposity and Bone Laboratory (MABlab) ULR 4490, Department of Rheumatology, Lille, France; 7 Department of Big Data in Health Science, School of Public Health and The Second Affiliated Hospital, Zhejiang University School of Medicine, Hangzhou, China; 8 Medical Research Council Human Genetics Unit, Medical Research Council Institute of Genetics & Molecular Medicine, University of Edinburgh, Edinburgh, UK; 9 Danish Institute for Advanced Study (DIAS), Epidemiology, Biostatistics and Biodemography, Department of Public Health, University of Southern Denmark, Odense, Denmark; 10 Cancer Research UK Edinburgh Centre, Medical Research Council Institute of Genetics and Cancer, University of Edinburgh, Edinburgh, UK; 11 Colon Cancer Genetics Group, Institute of Genetics and Cancer, University of Edinburgh, Edinburgh, UK; 12 Centre for Clinical Brain Sciences, University of Edinburgh, The Queen’s Medical Research Institute, Edinburgh BioQuarter, 47 Little France Crescent, Edinburgh, UK; 13 Edinburgh Cancer Research Centre, Institute of Genetics and Molecular Medicine, University of Edinburgh, Edinburgh, UK

**Keywords:** Genome-wide association studies, Bone, Machine learning

Correction to: *Nature Communications* 10.1038/s41467-024-55422-4, published online 2 January 2025

In the version of this article initially published, in the fourth paragraph of the “Sex sensitivity analysis for meta-GWAS in the white population” section, in the sentence now reading “Notably, we identified three sex-specific genes: *AKNA* and *WHRN* were associated with total hip BMFF in males only, while *VMPI* was associated with spine BMFF in females only,” *AKNA* originally appeared as “*ANKA*.” The error has been corrected in the HTML and PDF versions of the article.

