# Supplementary material for: Deep learning and genome-wide association meta-analyses of bone marrow adiposity in the UK Biobank
Source: Nat Commun. 2025 Jan 2;16:99. doi: 10.1038/s41467-024-55422-4 (PMC11697225; doi:10.1038/s41467-024-55422-4)
Supplement: Supplementary file 1 — Supplementary Information [file 41467_2024_55422_MOESM1_ESM.pdf]

SUPPLEMENTARY INFORMATION

Deep learning and genome-wide association meta-analyses of bone marrow adiposity in the UK Biobank

Wei Xu[1,2#], Ines Mesa-Eguiagaray[1#], David M Morris[2,3], Chengjia Wang[3,4], Calum D Gray[3], Samuel Sjöström[2], Giorgos Papanastasiou[3,5], Sammy Badr[6], Julien Paccou[6], Xue Li[7], Paul R. H. J. Timmers[9], Maria Timofeeva[8,9], Susan M. Farrington[10,11], Malcolm G. Dunlop[10,11], Scott IK Semple[2,3], Tom MacGillivray[12], Evropi Theodoratou[1,13*], and William P Cawthorn[2*]

This Supplementary Information file contains the following:

- **Supplementary Note 1**
- **Supplementary Figures 1-28**

Supplementary Note 1

GWAS in the white population

We used linkage disequilibrium score regression (LDSC: https://github.com/bulik/ldsc) to estimate genomic inflation and SNP-based heritability (h^2SNP). The SNP-based heritability (h^2_{SNP}) of GWAS in the first and the second batch, estimated through linkage disequilibrium score regression (LDSC) ranged from 18.63% to 28.04%. The LDSC intercepts were all close to 1, with λ_{GC} ranged from 1.056 to 1.111, suggesting that the observed inflation in genetic signal were consistent with polygenicity. LDSC showed high concordance of SNPs associations between the first and the second batch with r_g approximating 1 (Supplementary Data 10). The direction of effect size for the reported associations in each batch remained consistent.

Sex-specific meta-GWAS in the white population

We conducted GWAS meta-analyses stratified by sex in the unrelated white population for the four bone regions to investigate sex-specific BMFF-associated genetic variants (Supplementary Data 39-40).

In the femoral head, FUMA identified M:4/F:11 lead SNPs ($r^2 < 0.1$) and M:6/F:29 independent SNPs ($r^2 < 0.6$), residing in M:4/F:9 genomic risk loci, with I^2 <65% reached genome-wide significance ($P < 5 \times 10^{-8}$) in male and female groups respectively (Supplementary Data 39-40). LDSC results suggested that the signals were all consistent in polygenicity [Male: h^2_{SNP}=23.37% (SE=3.82%), intercept_meta=0.994 (SE=0.008), λ_{GC}_meta=1.065; Female: h^2_{SNP}=24.18% (SE=3.08%), intercept_meta=1.015 (SE=0.008), λ_{GC}_meta=1.086]. We found M:12/F:8 mapped genes in femoral head GWAS meta-analysis (Supplementary Data 41), in which *CDK5RAP1*, *SNTA1*, *CBFA2T2*, *NECAB3*, *C20orf144*, *ACTL10*, *E2F1* were male-specific and *LEPR*, *CCDC170*, *SLC14A2* were female specific.

In the total hip, we found that M:10/F:16 lead SNPs ($r^2 < 0.1$) and M:22/F:53 independent SNPs ($r^2 < 0.6$), residing in M:10/F:12 genomic risk loci, with I^2 <65% were associated at genome-wide significance ($P < 5 \times 10^{-8}$) in male and female groups respectively (Supplementary Data 39-40). Genomic inflation was moderate and consistent in polygenicity [Male: h^2_{SNP}=30.42% (SE=3.60%), intercept_meta=0.998 (SE=0.007), λ_{GC}_meta=1.080; Female: h^2_{SNP}=30.50% (SE=3.08%), intercept_meta=1.015 (SE=0.007), λ_{GC}_meta=1.111]. FUMA positional mapping found M:11/F:15 mapped genes in total hip GWAS meta-analysis (Supplementary Data 41).

In the femoral diaphysis, the findings indicated that M:14/F:12 lead SNPs ($r^2 < 0.1$) and M:32/F:47 independent SNPs ($r^2 < 0.6$), residing in M:9/F:7 genomic risk loci, with I^2 <65% reached genome-wide significance ($P < 5 \times 10^{-8}$) in male and female groups respectively (Supplementary Data 39-40). LDSC results suggested that most of the genomic inflation could be explained by polygenic signal [Male: h^2_{SNP}=27.49% (SE=3.45%), intercept_meta=1.002 (SE=0.007), λ_{GC}_meta=1.077; Female: h^2_{SNP}=32.63% (SE=3.51%), intercept_meta=1.008 (SE=0.008), λ_{GC}_meta=1.105]. FUMA identified M:13/F:12 mapped genes in femoral diaphysis GWAS meta-analysis (Supplementary Data 41).

In the spine, we found that M:10/F:17 lead SNPs ($r^2 < 0.1$) and M:31/F:46 independent SNPs ($r^2 < 0.6$), residing in M:9/F:12 genomic risk loci, with I^2 <65% was associated at genome-wide significance ($P < 5 \times 10^{-8}$) in male and female groups respectively (Supplementary Data 39-40). LDSC results suggested that the signals were all consistent in polygenicity [Male: h^2_{SNP}=26.22% (SE=3.83%), intercept_meta=0.998 (SE=0.009), λ_{GC}_meta=1.068; Female: h^2_{SNP}=21.50% (SE=3.59%),

intercept_meta=1.021 (SE=0.008), λ_{GC}_meta=1.083]. We identified M:24/F:31 mapped genes in spine GWAS meta-analysis (Supplementary Data 41).

Supplementary Figures

SUPPLEMENTARY FIGURE 1

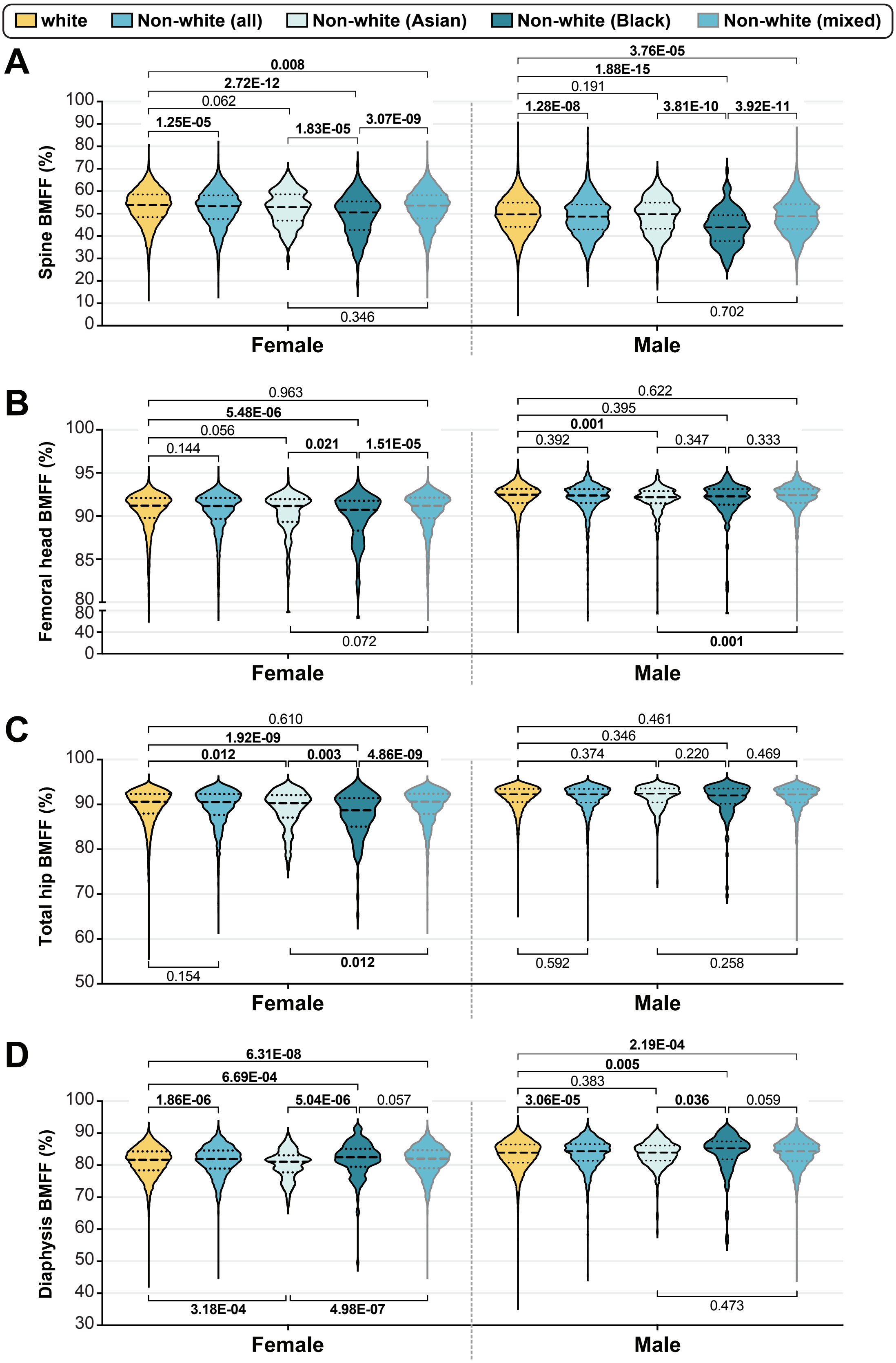

Supplementary Figure 1 – BMFF of the spine and femoral diaphysis differs between white and non-white participants.

(A-D) BMFF (%) of the spine (A), femoral head (B), total hip (C) and femoral diaphysis (D) for white and non-white females and males, as indicated. Data are presented as violin plots, with the median shown as a dashed horizontal line and the 25% and 75% as dotted horizontal lines, for the following numbers of participants: spine – white female (n=21111), non-white female, (n=3379), Asian female (n=274), Black female (n=167), mixed female (n=2938), white male (n=20093), non-white male (n=2988), Asian male (n=390), Black male (n=129), mixed male (n=2469); femoral head – white female (n=20536), non-white female (n=3211), Asian female (n=247), Black female (n=153), mixed female (n=2811), white male (n=18045), non-white male (n=2722), Asian male (n=381), Black male (n=117), mixed male (n=2224); total hip – white female (n=20890), non-white female (n=3346), Asian female (n=268), Black female (n=164), mixed female (n=2914), white male (n=17504), non-white male (n=2701), Asian male (n=382), Black male (n=132), mixed male (n=2187); diaphysis – white female (n=20576), non-white female (n=3265), Asian female (n=256), Black female (n=167), mixed female (n=2842), white male (n=16937), non-white male (n=2579), Asian male (n=361), Black male (n=118), mixed male (n=2100). The numbers per group may differ slightly from those shown in Supplementary Data 7, because data for BMI and/or BMD may not have been available for all participants. Within each site and sex, differences between white and non-white participants were assessed by comparing rank-normalized BMFF values using multivariate ANOVA, controlling for BMI and age at imaging. Adjusted *P* values for each pairwise comparison are shown. Adjusted *P* with further controlling for BMD are shown in Supplementary Data 7.

SUPPLEMENTARY FIGURE 2

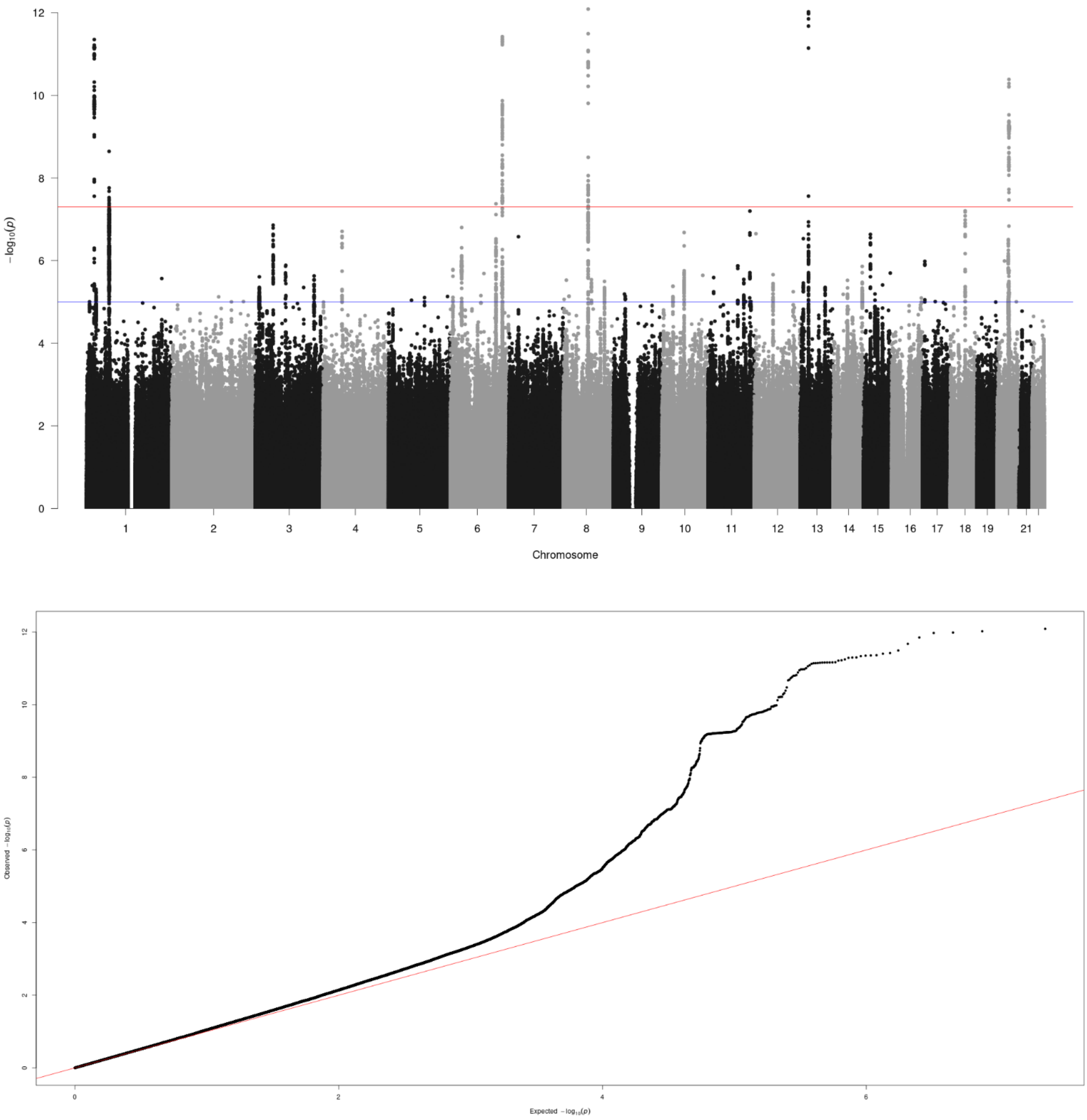

Supplementary Figure 2 – Manhattan plot and QQ plot of the GWAS results for the first batch in white participants for femoral head (N=24,787). The Manhattan plot (top) shows the genomic position of each variant on the x-axis and the GWAS negative log10-transformed P-value on the y-axis. Independent lead variants of each locus are annotated by a diamond. The horizontal red line indicates the genome-wide significance threshold that corresponds to a P-value of 5×10^{-8}, while the horizontal blue indicates the suggestive threshold of 1×10^{-5}. The QQ plot (bottom) shows the observed and expected P-value distribution of the genetic variants.

SUPPLEMENTARY FIGURE 3

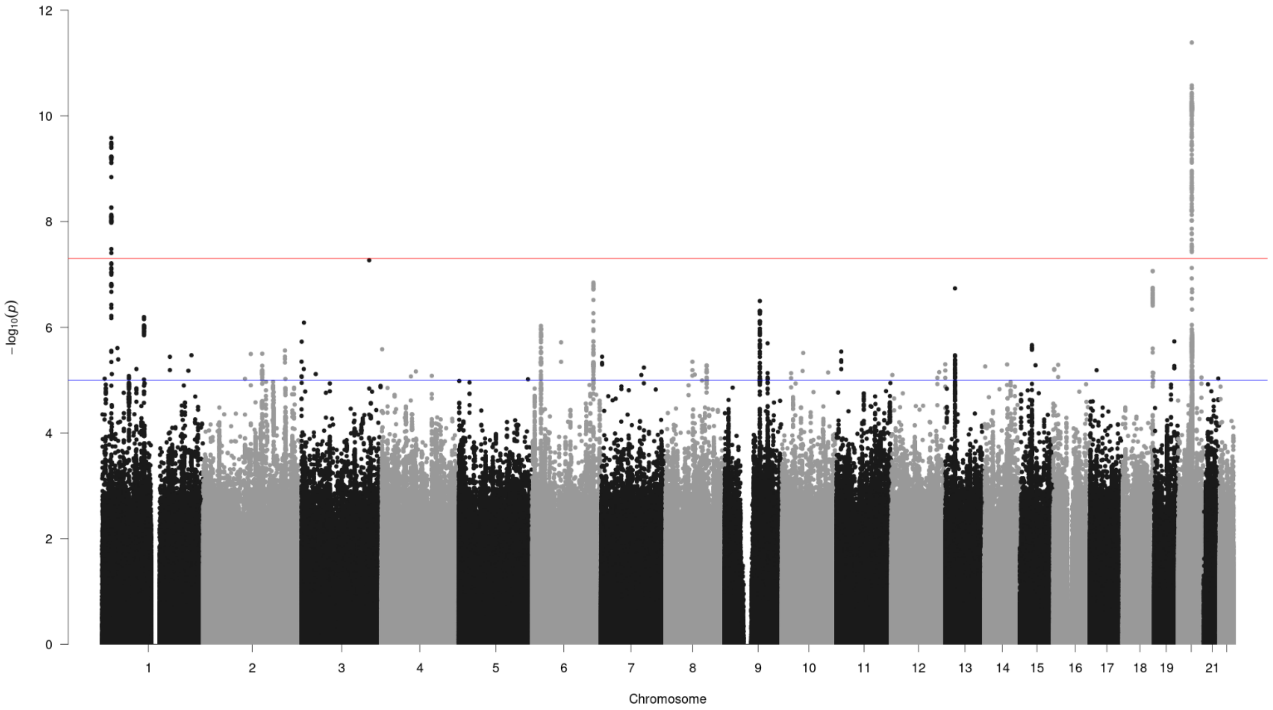

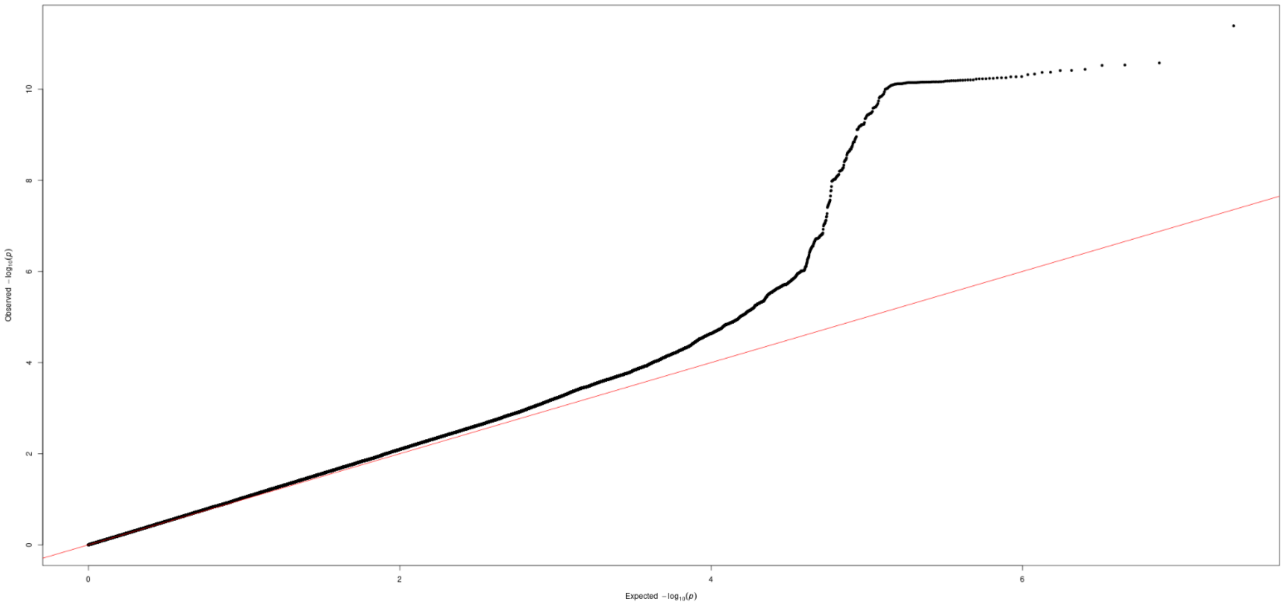

Supplementary Figure 3 – Manhattan plot and QQ plot of the GWAS results for the second batch in white participants for femoral head (N=13,794). The Manhattan plot (top) shows the genomic position of each variant on the x-axis and the GWAS negative log10-transformed P-value on the y-axis. Independent lead variants of each locus are annotated by a diamond. The horizontal red line indicates the genome-wide significance threshold that corresponds to a P-value of 5×10^{-8}, while the horizontal blue indicates the suggestive threshold of 1×10^{-5}. The QQ plot (bottom) shows the observed and expected P-value distribution of genetic variants.

SUPPLEMENTARY FIGURE 4

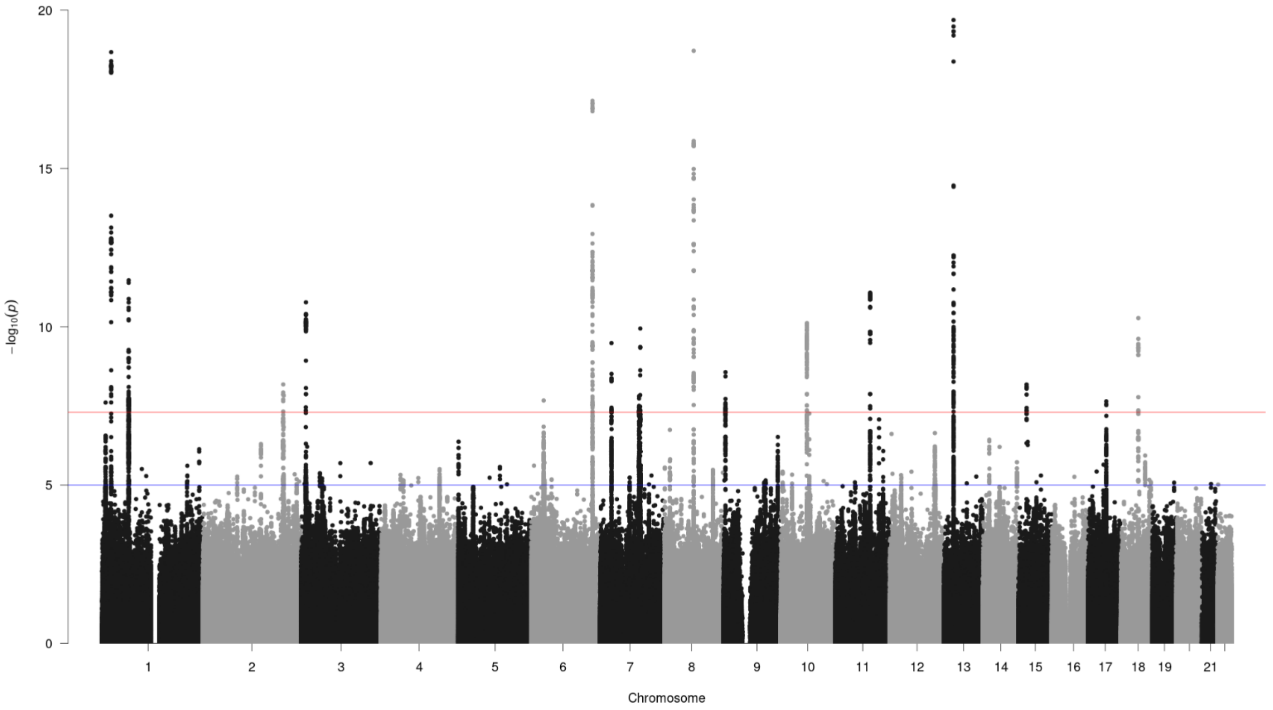

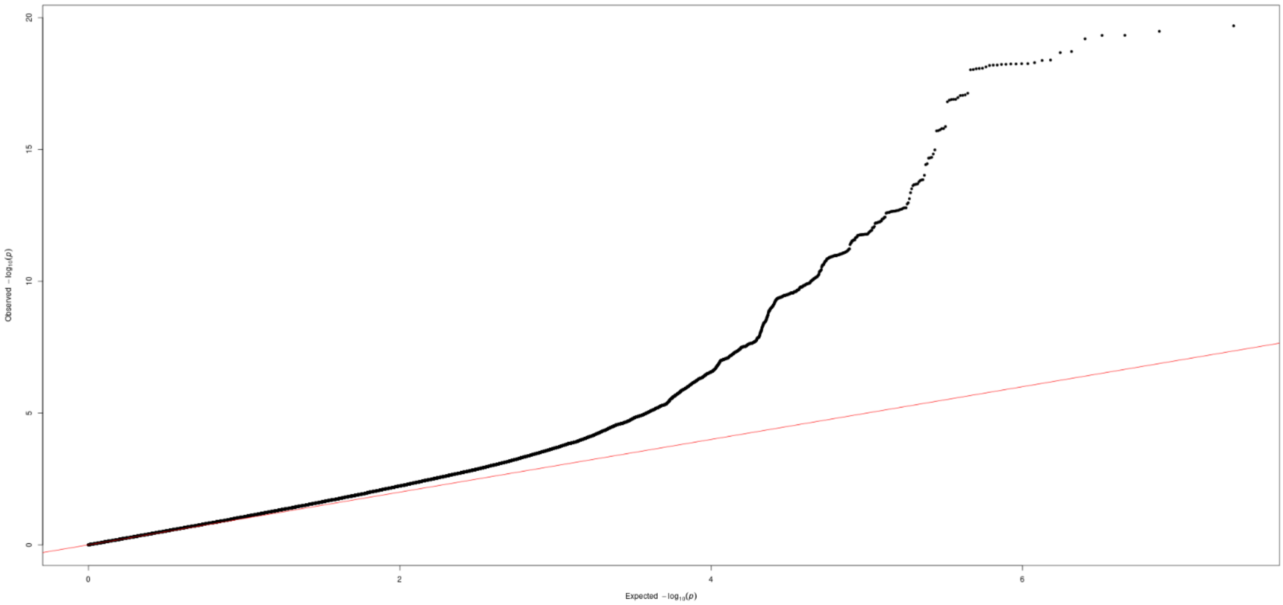

Supplementary Figure 4 – Manhattan plot and QQ plot of the GWAS results for the first batch in white participants for total hip (N=24,366). The Manhattan plot (top) shows the genomic position of each variant on the x-axis and the GWAS negative log10-transformed P-value on the y-axis. Independent lead variants of each locus are annotated by a diamond. The horizontal red line indicates the genome-wide significance threshold that corresponds to a P-value of 5×10^{-8}, while the horizontal blue indicates the suggestive threshold of 1×10^{-5}. The QQ plot (bottom) shows the observed and expected P-value distribution of genetic variants.

SUPPLEMENTARY FIGURE 5

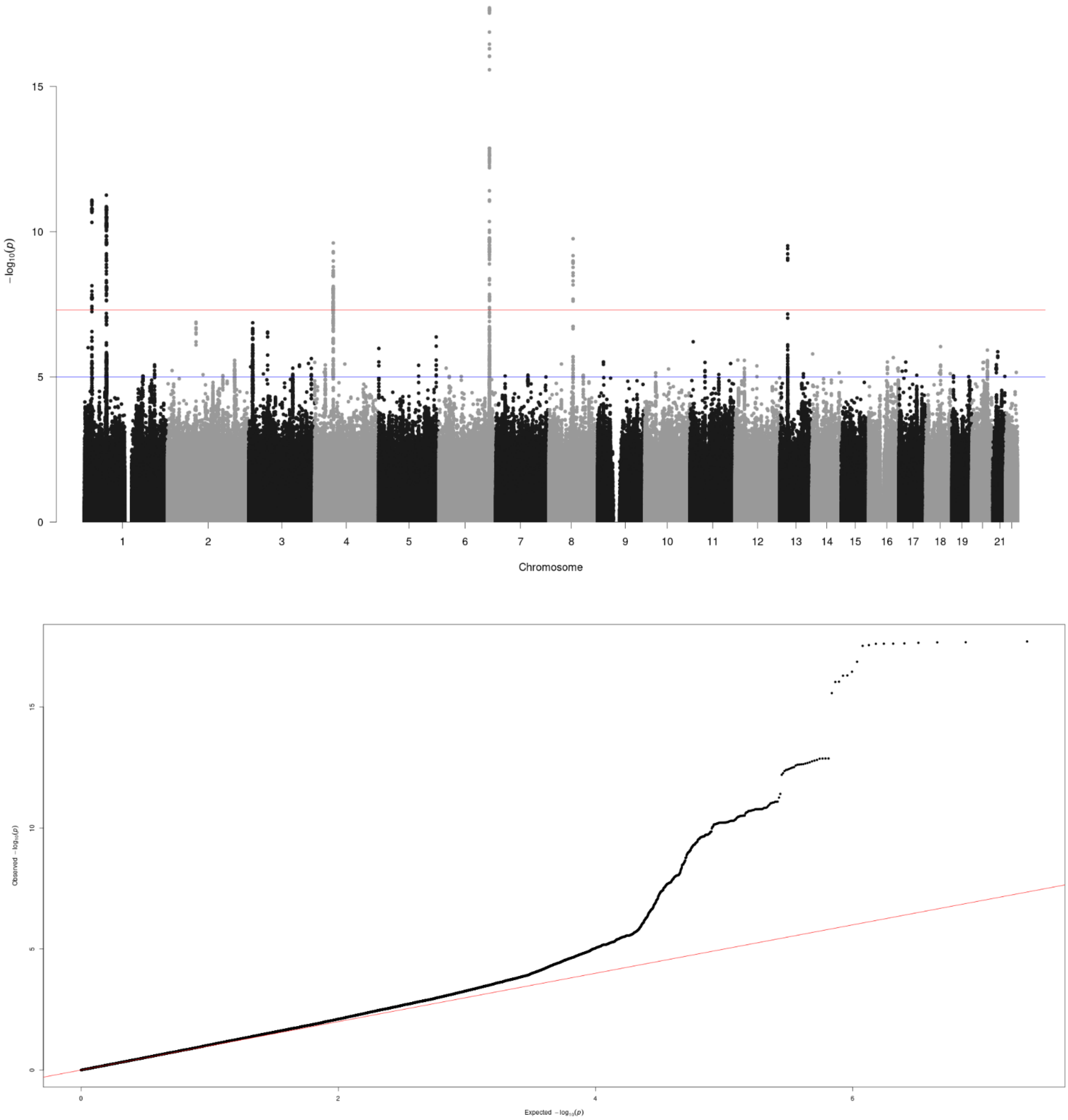

Supplementary Figure 5 – Manhattan plot and QQ plot of the GWAS results for the second batch in white participants for total hip (N=14,028). The Manhattan plot (top) shows the genomic position of each variant on the x-axis and the GWAS negative log10-transformed P-value on the y-axis. Independent lead variants of each locus are annotated by a diamond. The horizontal red line indicates the genome-wide significance threshold that corresponds to a P-value of 5×10^{-8}, while the horizontal blue indicates the suggestive threshold of 1×10^{-5}. The QQ plot (bottom) shows the observed and expected P-value distribution of genetic variants.

SUPPLEMENTARY FIGURE 6

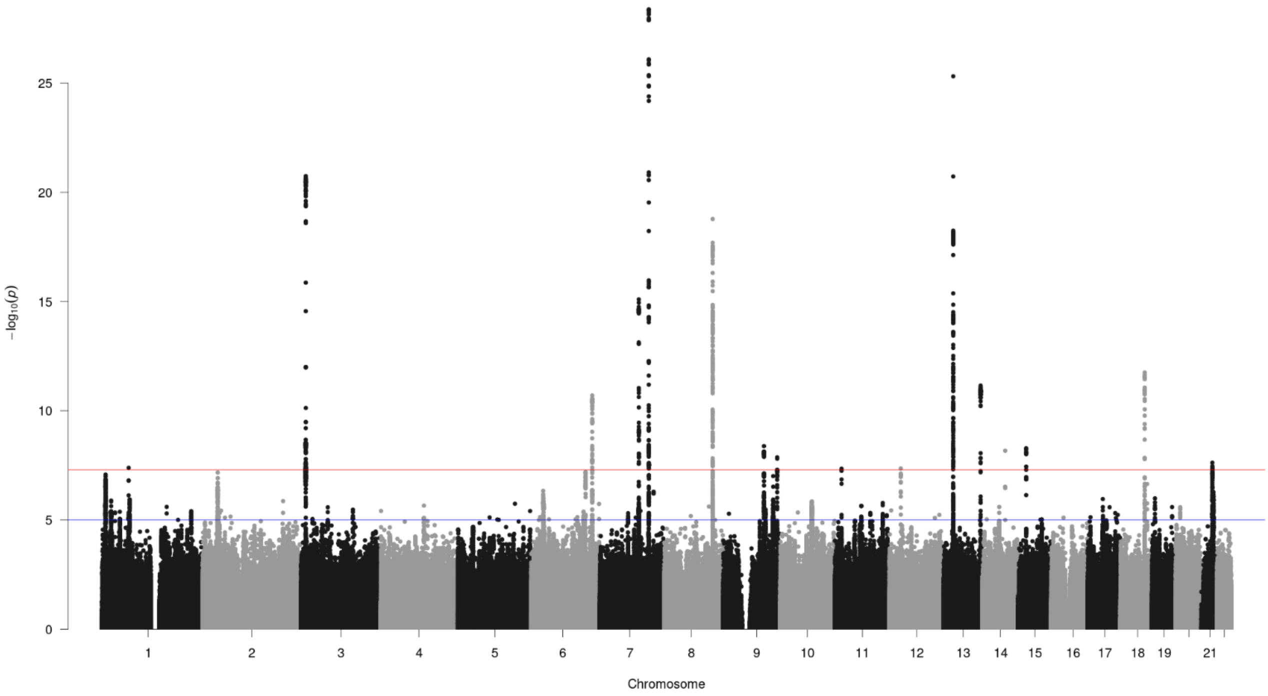

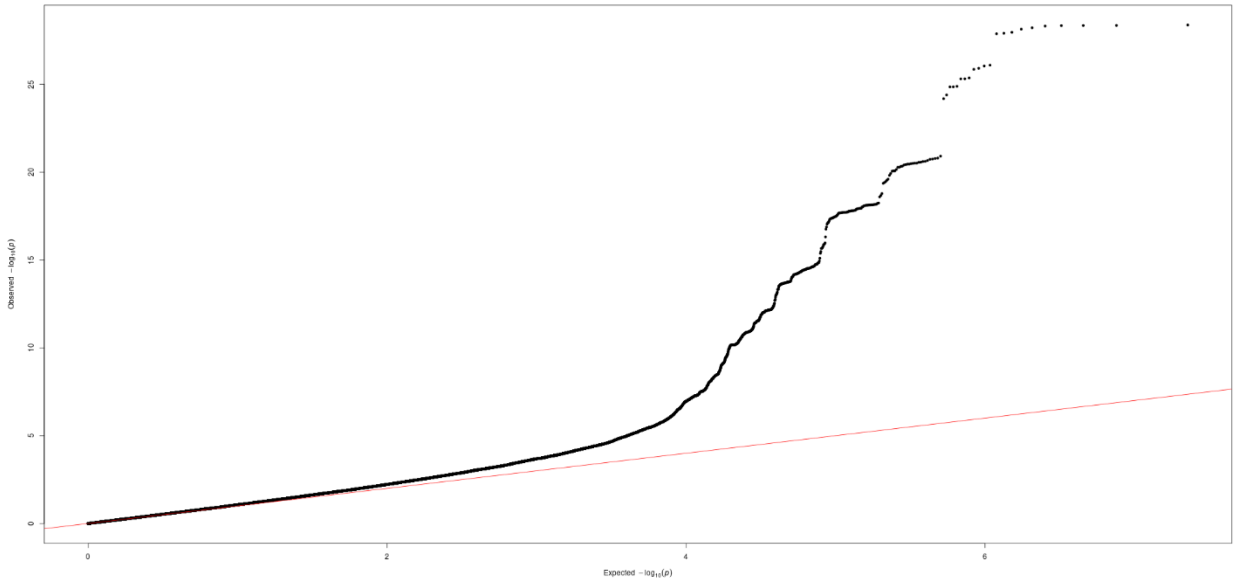

Supplementary Figure 6 – Manhattan plot and QQ plot of the GWAS results for the first batch in white participants for diaphysis (N=24,175). The Manhattan plot (top) shows the genomic position of each variant on the x-axis and the GWAS negative log10-transformed P-value on the y-axis. Independent lead variants of each locus are annotated by a diamond. The horizontal red line indicates the genome-wide significance threshold that corresponds to a P-value of 5×10^{-8}, while the horizontal blue indicates the suggestive threshold of 1×10^{-5}. The QQ plot (bottom) shows the observed and expected P-value distribution of genetic variants.

SUPPLEMENTARY FIGURE 7

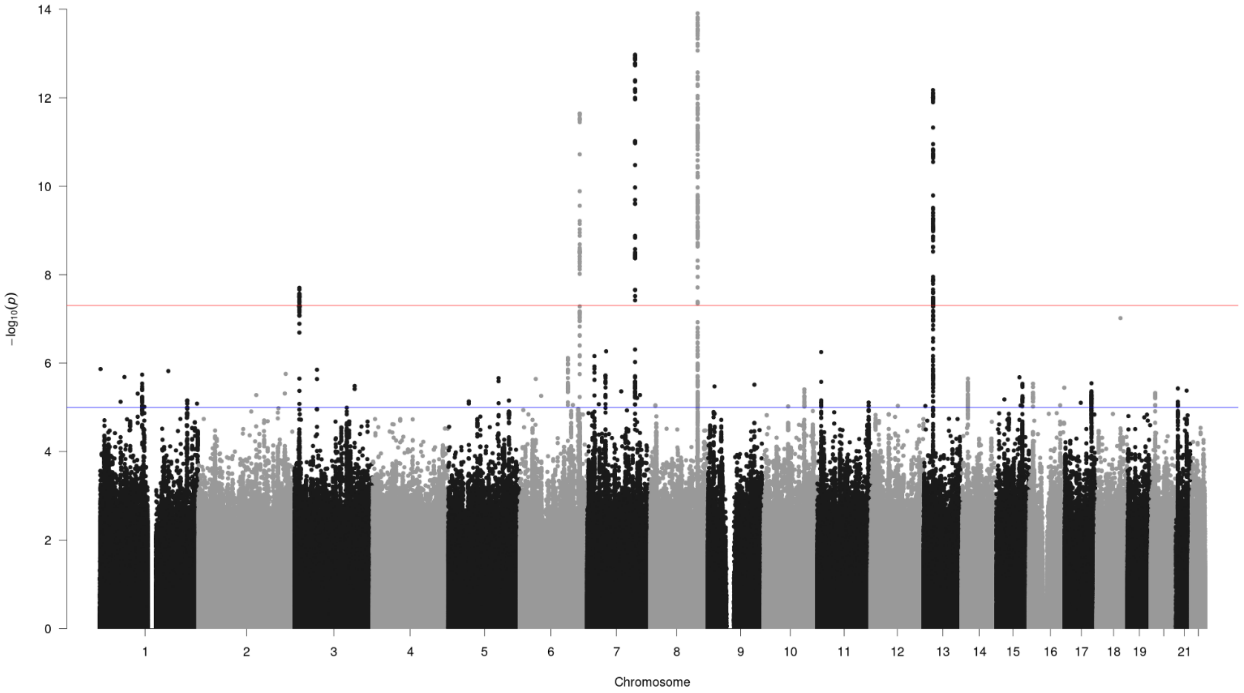

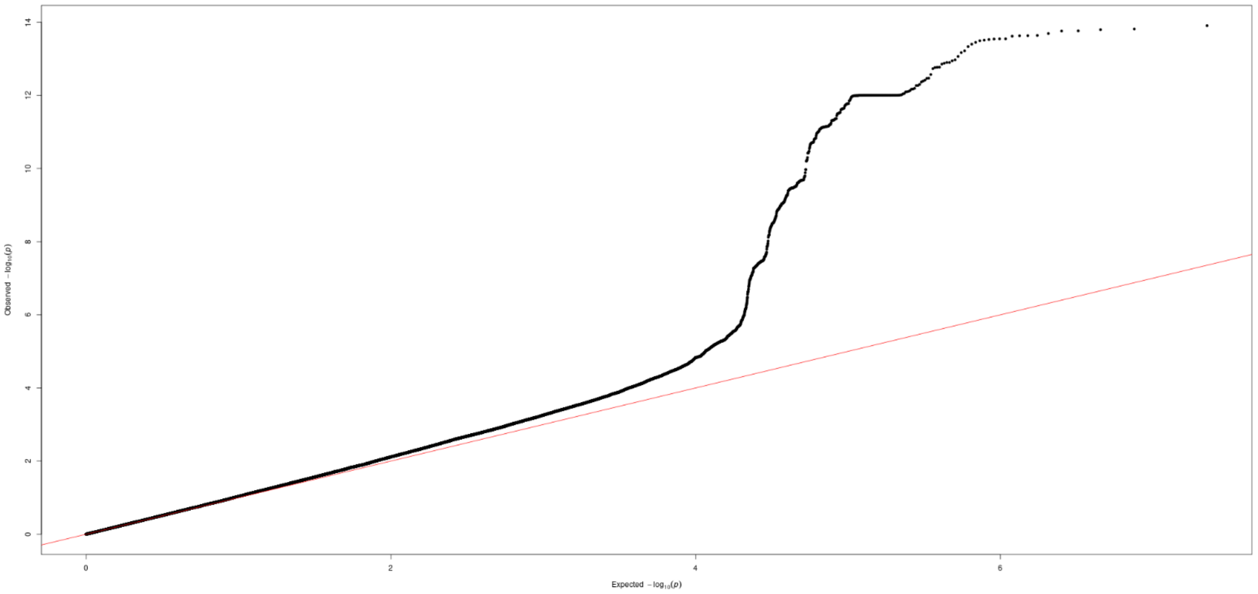

Supplementary Figure 7 – Manhattan plot and QQ plot of the GWAS results for the second batch in white participants for diaphysis (N=13,338). The Manhattan plot (top) shows the genomic position of each variant on the x-axis and the GWAS negative log10-transformed P-value on the y-axis. Independent lead variants of each locus are annotated by a diamond. The horizontal red line indicates the genome-wide significance threshold that corresponds to a P-value of 5×10^{-8}, while the horizontal blue indicates the suggestive threshold of 1×10^{-5}. The QQ plot (bottom) shows the observed and expected P-value distribution of genetic variants.

SUPPLEMENTARY FIGURE 8

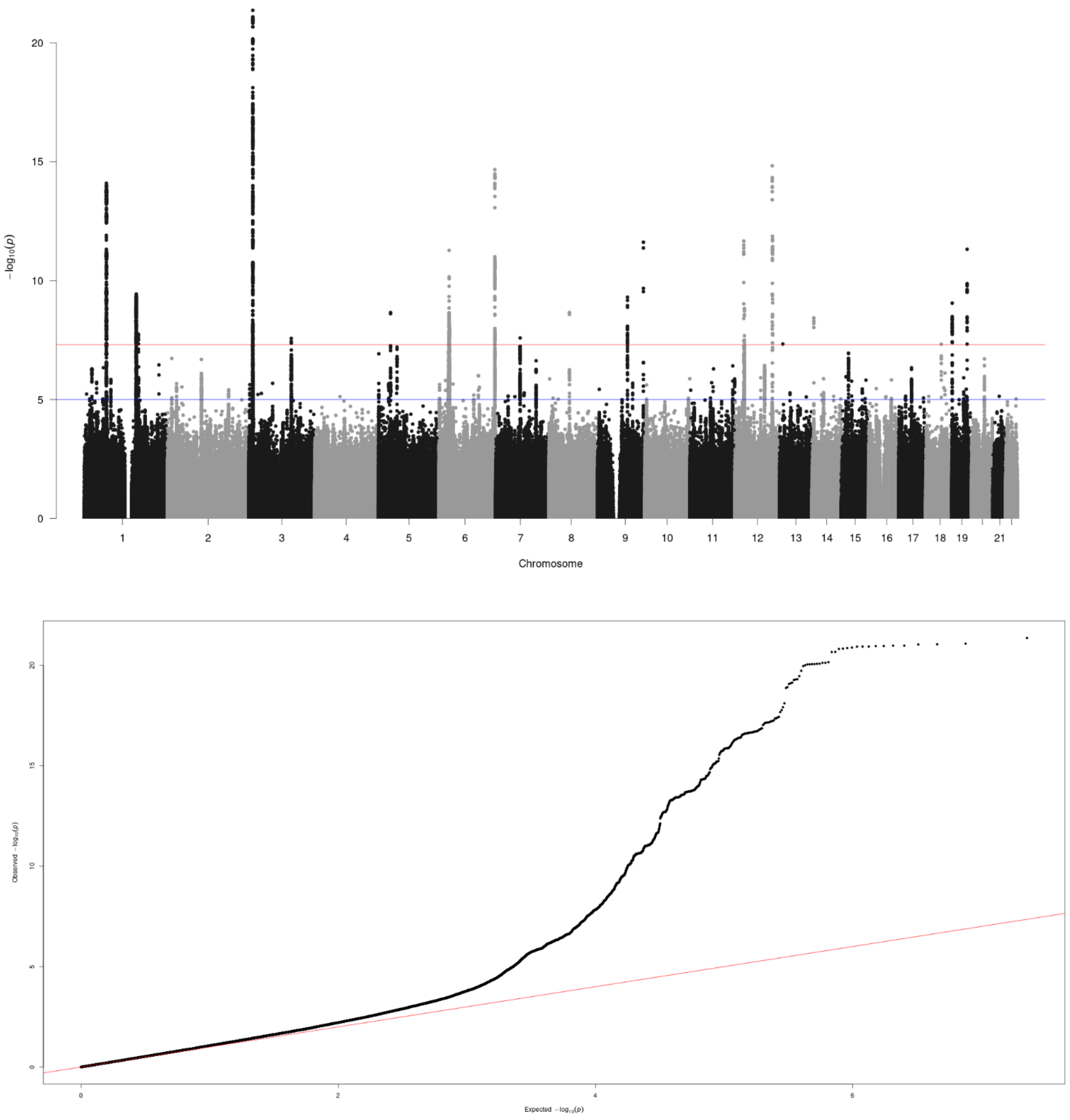

Supplementary Figure 8 – Manhattan plot and QQ plot of the GWAS results for the first batch in white participants for spine (N=26,329). The Manhattan plot (top) shows the genomic position of each variant on the x-axis and the GWAS negative log10-transformed P-value on the y-axis. Independent lead variants of each locus are annotated by a diamond. The horizontal red line indicates the genome-wide significance threshold that corresponds to a P-value of 5×10^{-8}, while the horizontal blue indicates the suggestive threshold of 1×10^{-5}. The QQ plot (bottom) shows the observed and expected P-value distribution of genetic variants.

SUPPLEMENTARY FIGURE 9

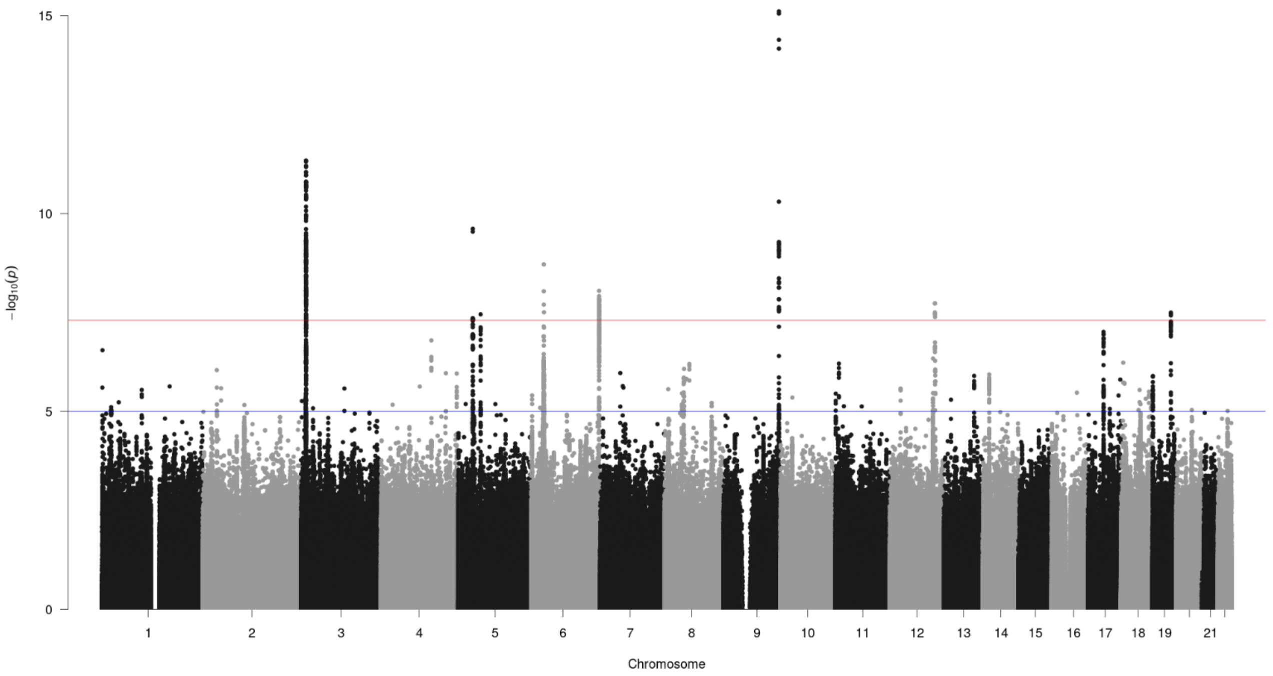

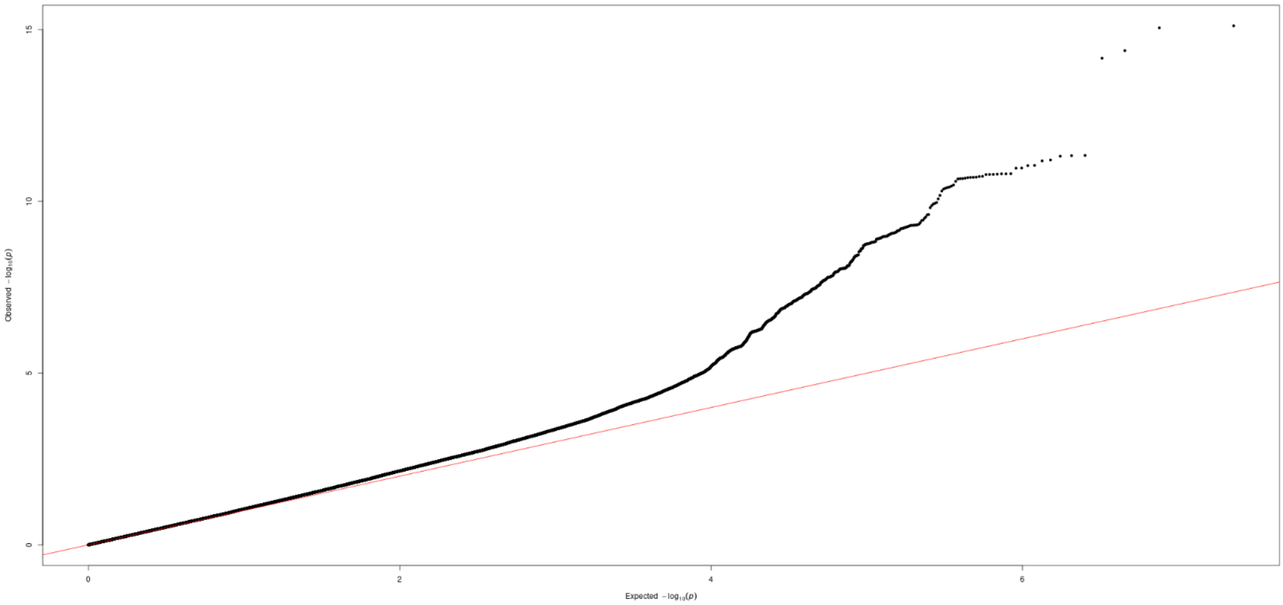

Supplementary Figure 9 – Manhattan plot and QQ plot of the GWAS results for the second batch in white participants for spine (N=14,875). The Manhattan plot (top) shows the genomic position of each variant on the x-axis and the GWAS negative log10-transformed P-value on the y-axis. Independent lead variants of each locus are annotated by a diamond. The horizontal red line indicates the genome-wide significance threshold that corresponds to a P-value of 5×10^{-8}, while the horizontal blue indicates the suggestive threshold of 1×10^{-5}. The QQ plot (bottom) shows the observed and expected P-value distribution of genetic variants.

SUPPLEMENTARY FIGURE 10

A - subcutaneous adipose tissue

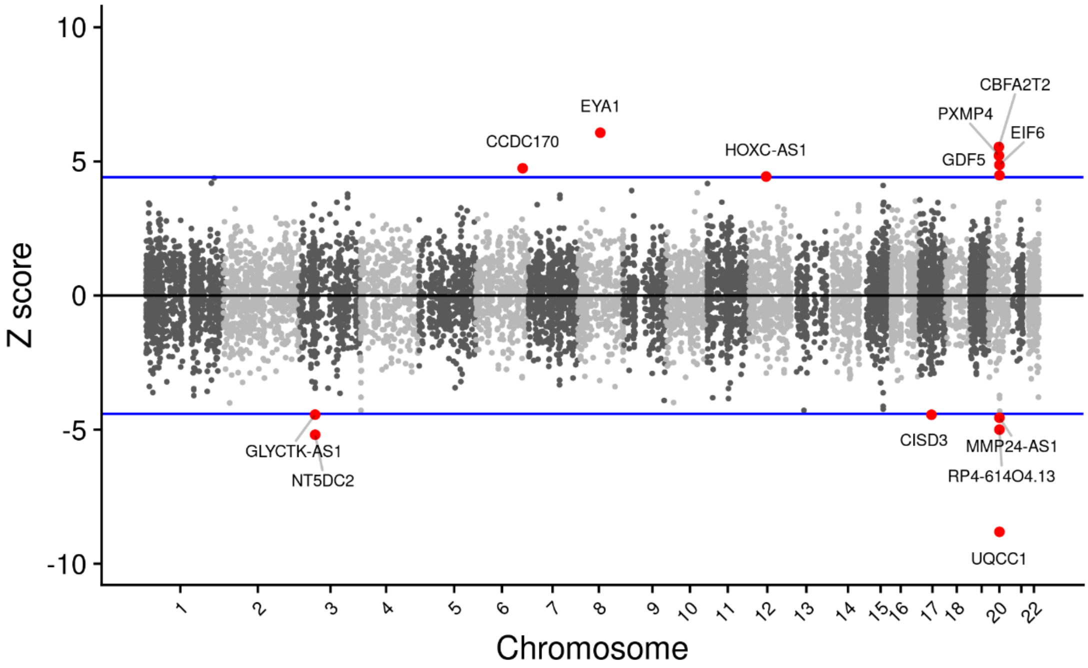

B - visceral (omentum) adipose tissue

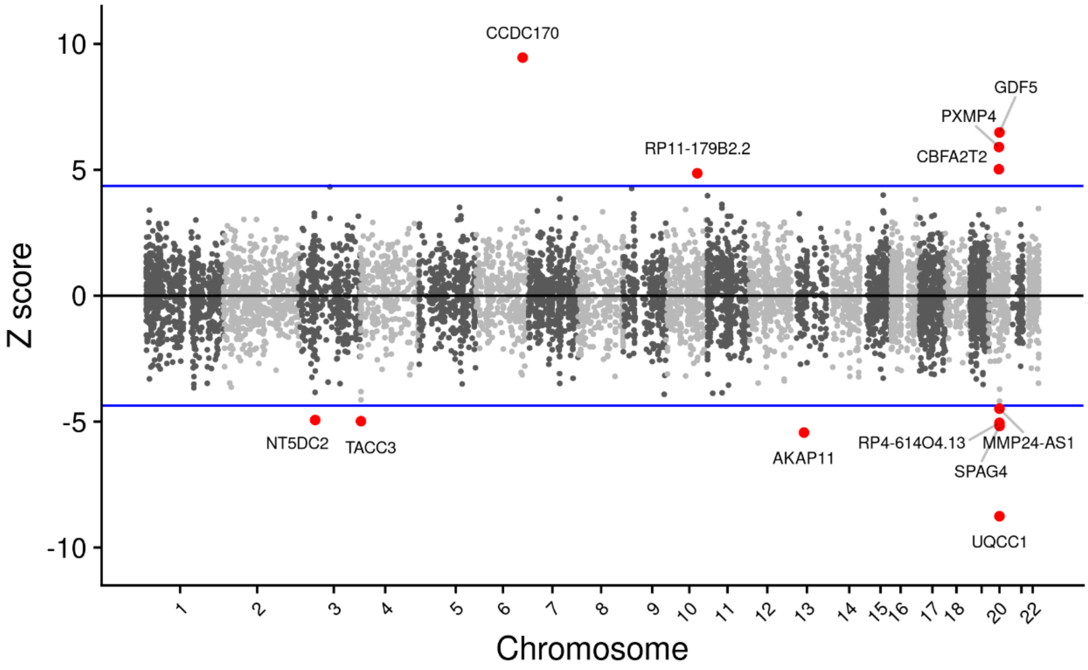

C – skeletal muscle tissue

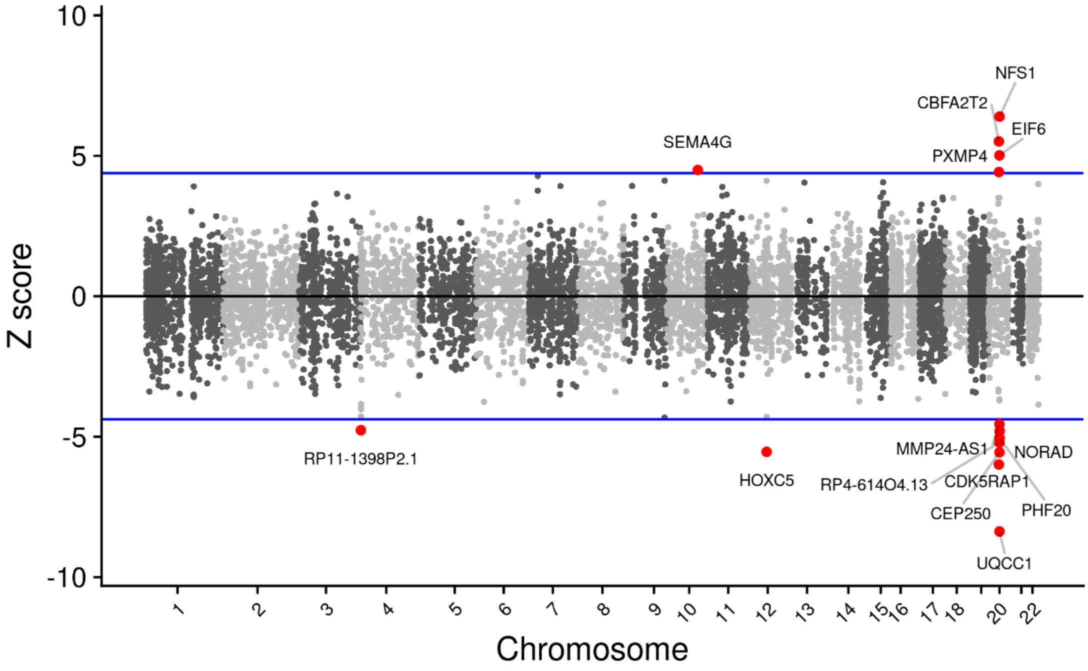

Supplementary Figure 10 – Manhattan-style Z-score plot of gene associations with BMFF in the TWAS based on the meta-GWAS-white for femoral head. TWAS associations across A - subcutaneous adipose tissue; B - visceral (omentum) adipose tissue; C – skeletal muscle tissue. Each dot represents a TWAS signal. The x-axis corresponds to the chromosomal position of the genes and the y-axis corresponds to the Z-score value representing the predicted expression level of each gene in BMFF. Blue lines indicate the transcriptome-wide significance threshold (Bonferroni-adjusted p value). The names of statistically significant genes are labeled on the corresponding dots. For genes on the top part of the graph, increased expression was associated with increased BMFF, while expression of the genes on the bottom part of the plot showed an inverse association.

SUPPLEMENTARY FIGURE 11

A - subcutaneous adipose tissue

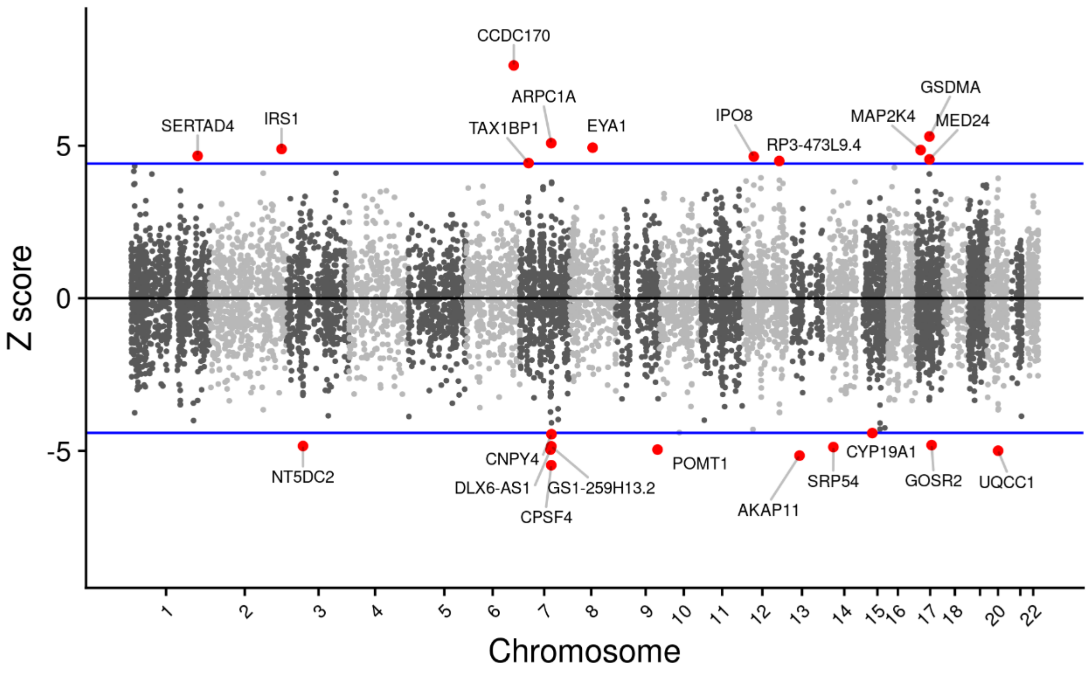

B - visceral (omentum) adipose tissue

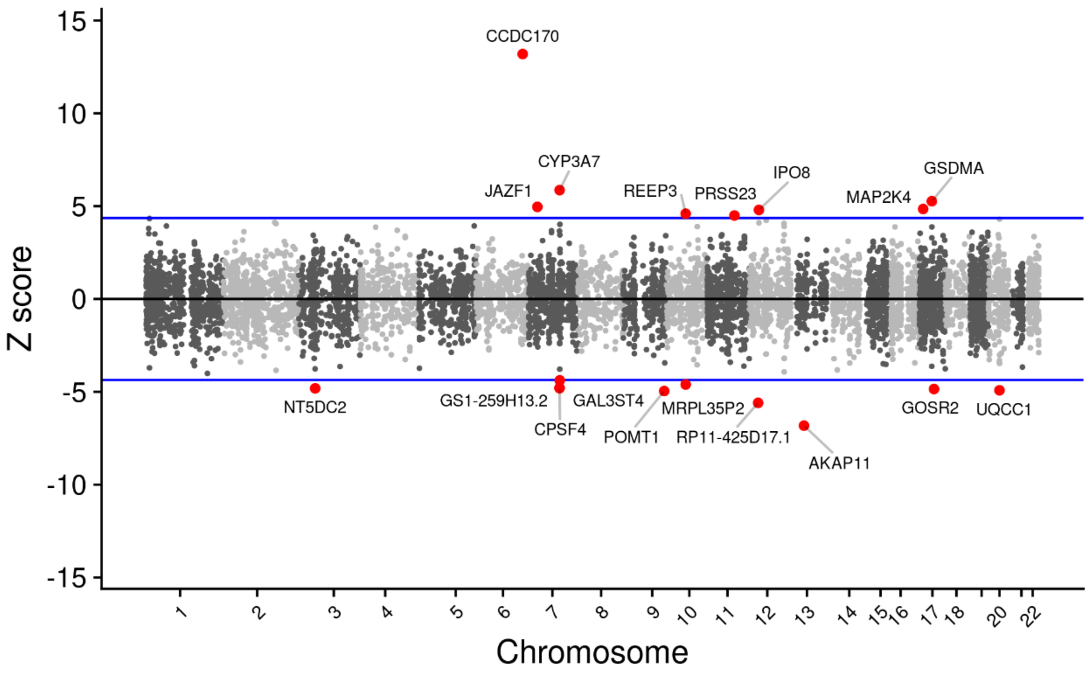

C – skeletal muscle tissue

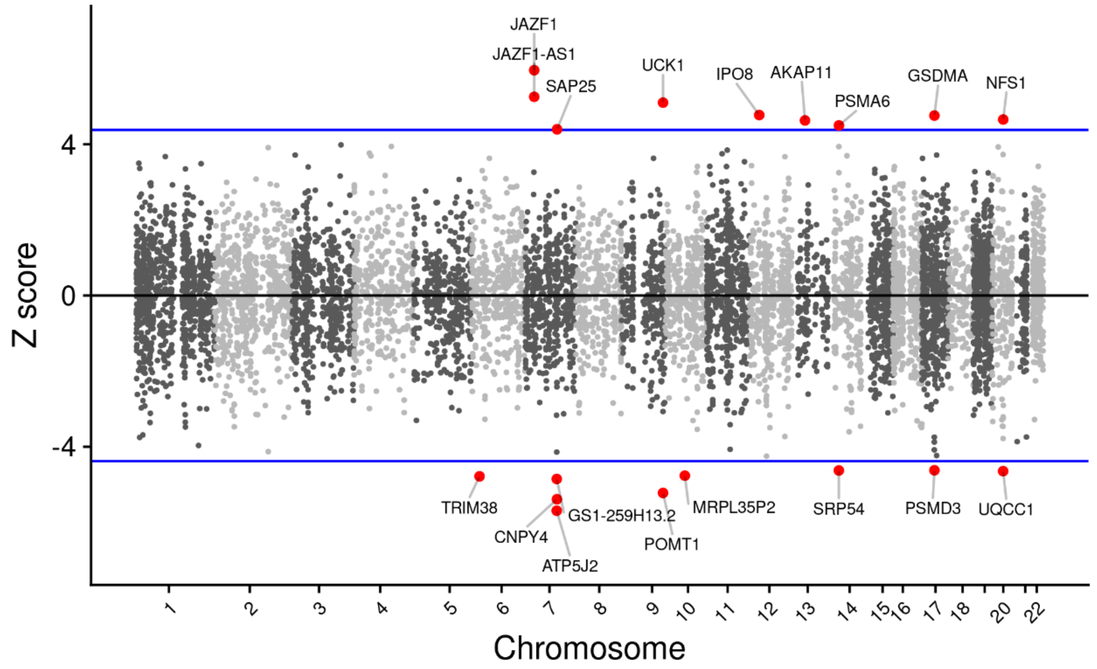

Supplementary Figure 11 – Manhattan-style Z-score plot of gene associations with BMFF in the TWAS based on the meta-GWAS-white for total hip. TWAS associations across A - subcutaneous adipose tissue; B - visceral (omentum) adipose tissue; C – skeletal muscle tissue. Each dot represents a TWAS signal. The x-axis corresponds to the chromosomal position of the genes and the y-axis corresponds to the Z-score value representing the predicted expression level of each gene in BMFF. Blue lines indicate the transcriptome-wide significance threshold (Bonferroni-adjusted p value). The names of statistically significant genes are labeled on the corresponding dots. For genes on the top part of the graph, increased expression was associated with increased BMFF, while expression of the genes on the bottom part of the plot showed an inverse association.

SUPPLEMENTARY FIGURE 12

A - subcutaneous adipose tissue

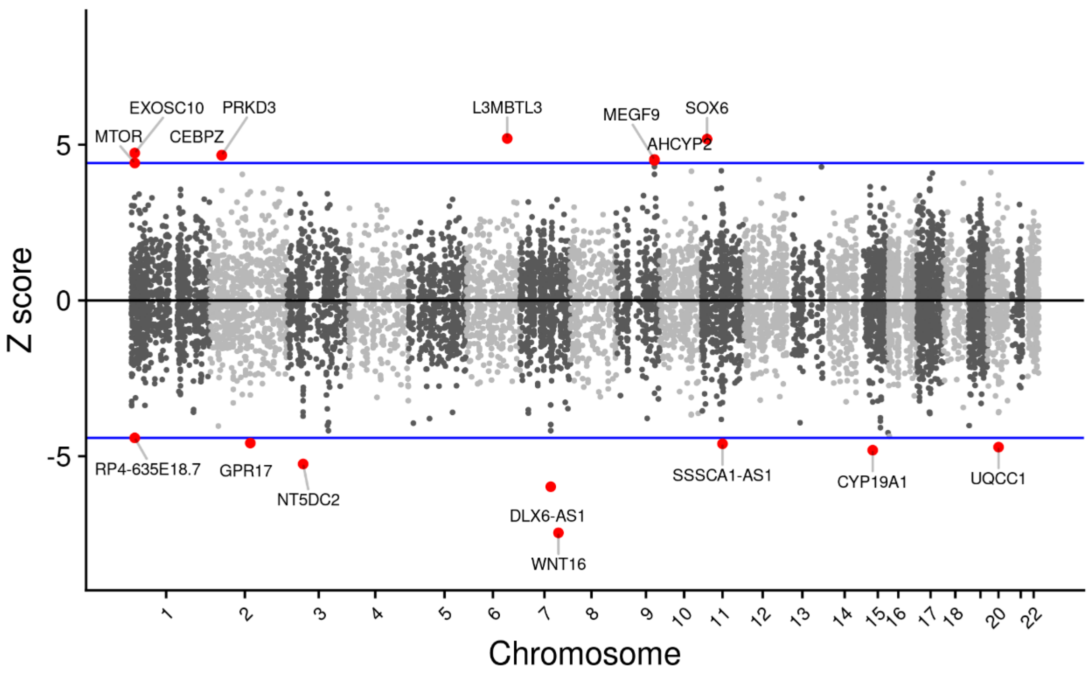

B - visceral (omentum) adipose tissue

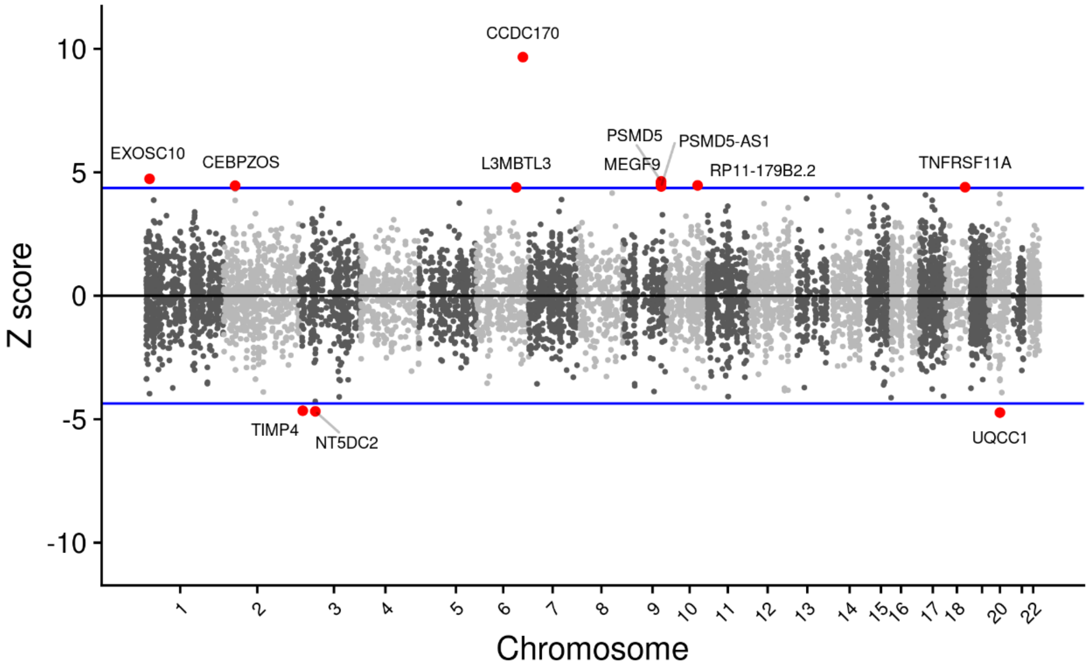

C – skeletal muscle tissue

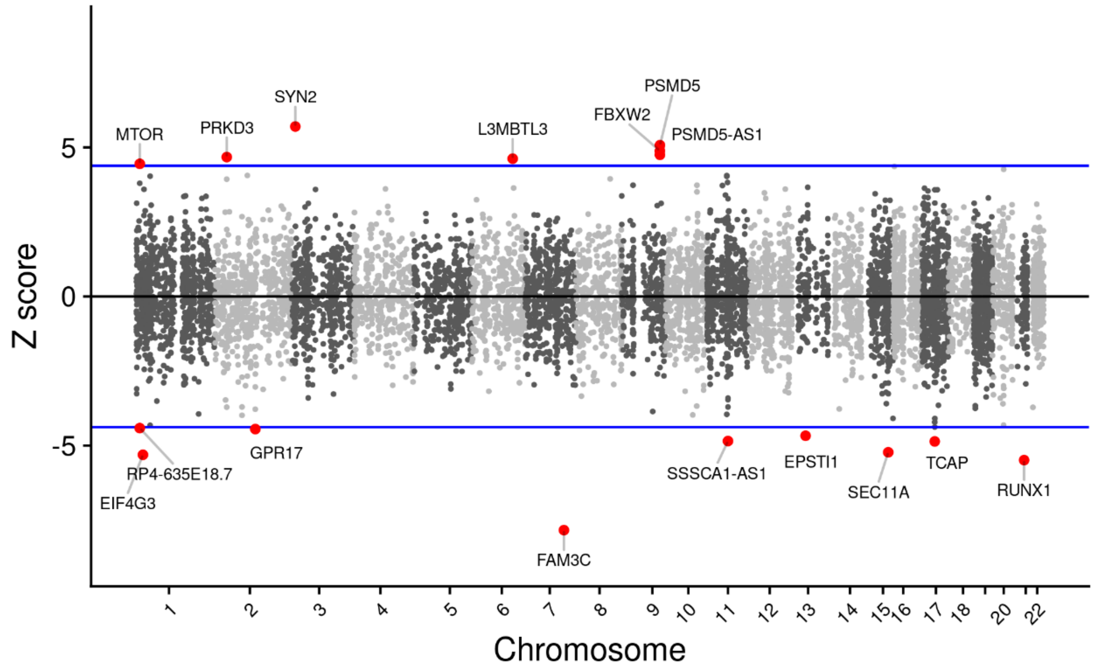

Supplementary Figure 12 – Manhattan-style Z-score plot of gene associations with BMFF in the TWAS based on the meta-GWAS-white for diaphysis. TWAS associations across A - subcutaneous adipose tissue; B - visceral (omentum) adipose tissue; C – skeletal muscle tissue. Each dot represents a TWAS signal. The x-axis corresponds to the chromosomal position of the genes and the y-axis corresponds to the Z-score value representing the predicted expression level of each gene in BMFF. Blue lines indicate the transcriptome-wide significance threshold (Bonferroni-adjusted p value). The names of statistically significant genes are labeled on the corresponding dots. For genes on the top part of the graph, increased expression was associated with increased BMFF, while expression of the genes on the bottom part of the plot showed an inverse association.

SUPPLEMENTARY FIGURE 13

A - subcutaneous adipose tissue

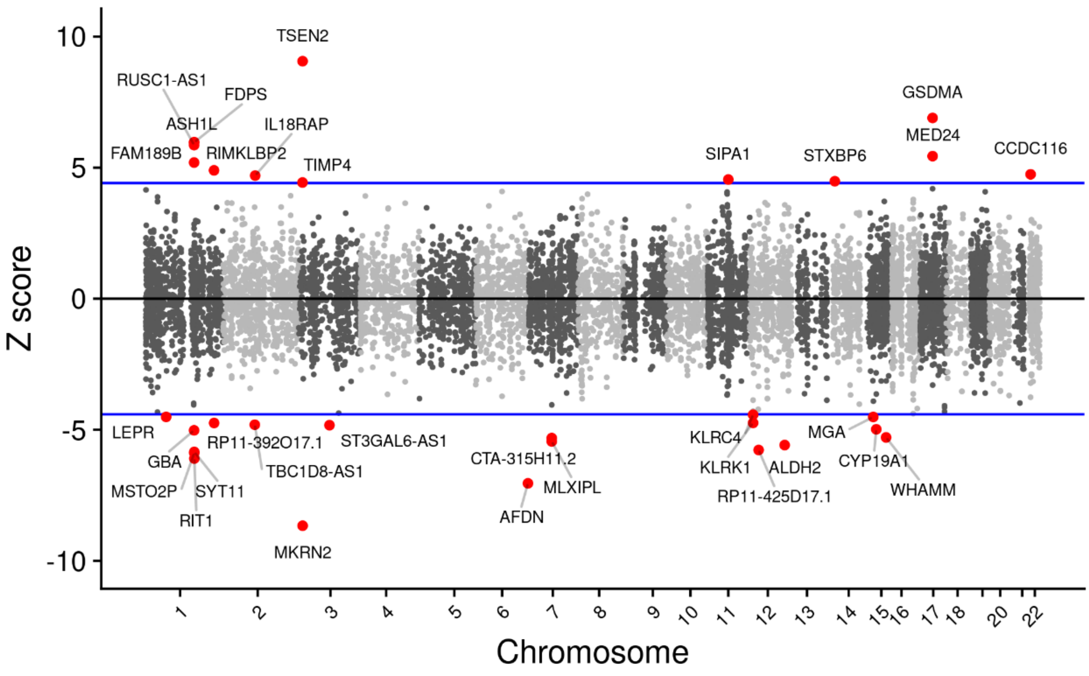

B - visceral (omentum) adipose tissue

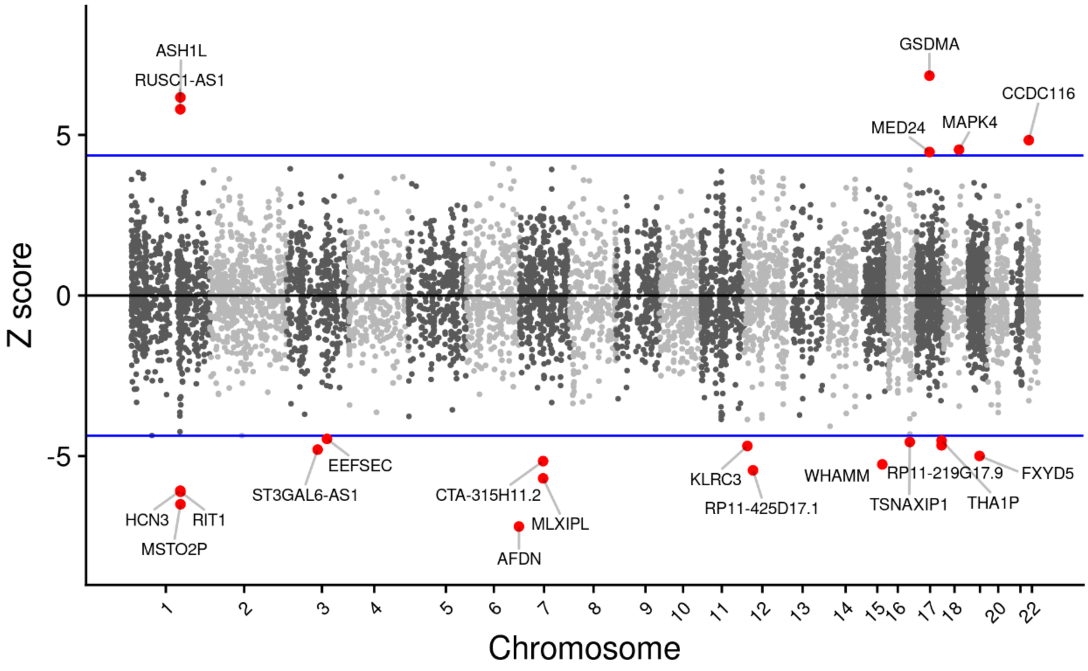

C – skeletal muscle tissue

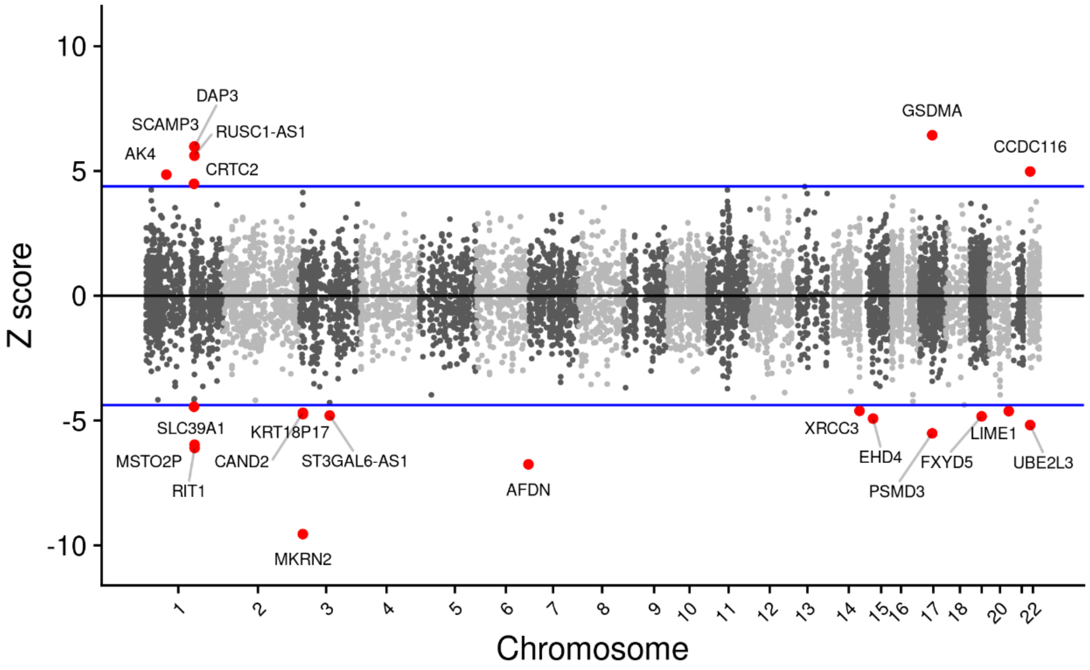

Supplementary Figure 13 – Manhattan-style Z-score plot of gene associations with BMFF in the TWAS based on the meta-GWAS-white for spine. TWAS associations across A - subcutaneous adipose tissue; B - visceral (omentum) adipose tissue; C – skeletal muscle tissue. Each dot represents a TWAS signal. The x-axis corresponds to the chromosomal position of the genes and the y-axis corresponds to the Z-score value representing the predicted expression level of each gene in BMFF. Blue lines indicate the transcriptome-wide significance threshold (Bonferroni-adjusted p value). The names of statistically significant genes are labeled on the corresponding dots. For genes on the top part of the graph, increased expression was associated with increased BMFF, while expression of the genes on the bottom part of the plot showed an inverse association.

SUPPLEMENTARY FIGURE 14

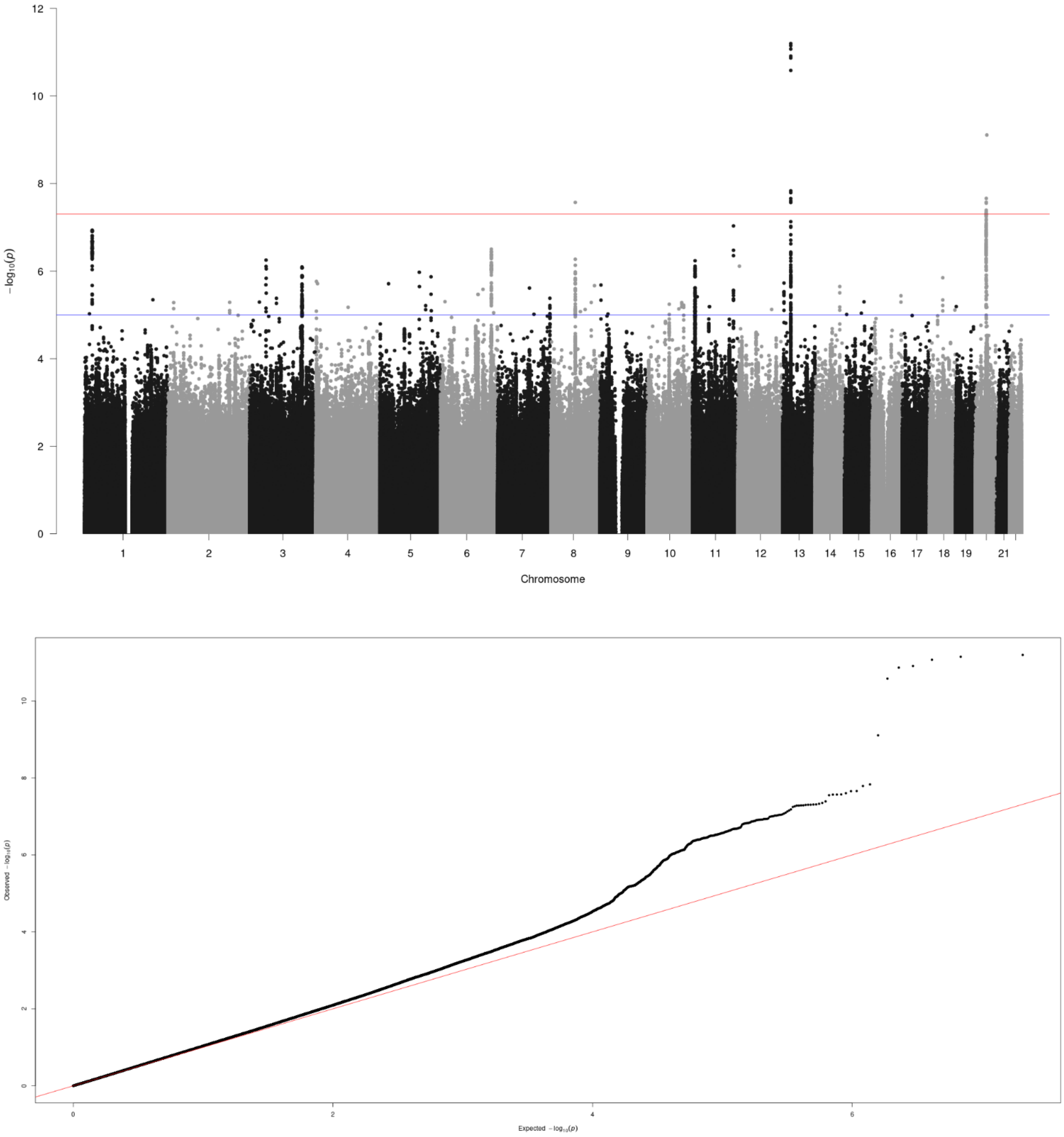

Supplementary Figure 14 – Manhattan plot of meta-GWAS results combining the first and second batch for femoral head in males (N=18,045). The Manhattan plot (top) shows the genomic position of each variant on the x-axis and the GWAS negative log10-transformed P-value on the y-axis. Independent lead variants of each locus are annotated by a diamond. The horizontal red line indicates the genome-wide significance threshold that corresponds to a P-value of 5×10^{-8}, while the horizontal blue indicates the suggestive threshold of 1×10^{-5}. The QQ plot (bottom) shows the observed and expected P-value distribution of genetic variants in the meta-GWAS for Femoral head in Males.

SUPPLEMENTARY FIGURE 15

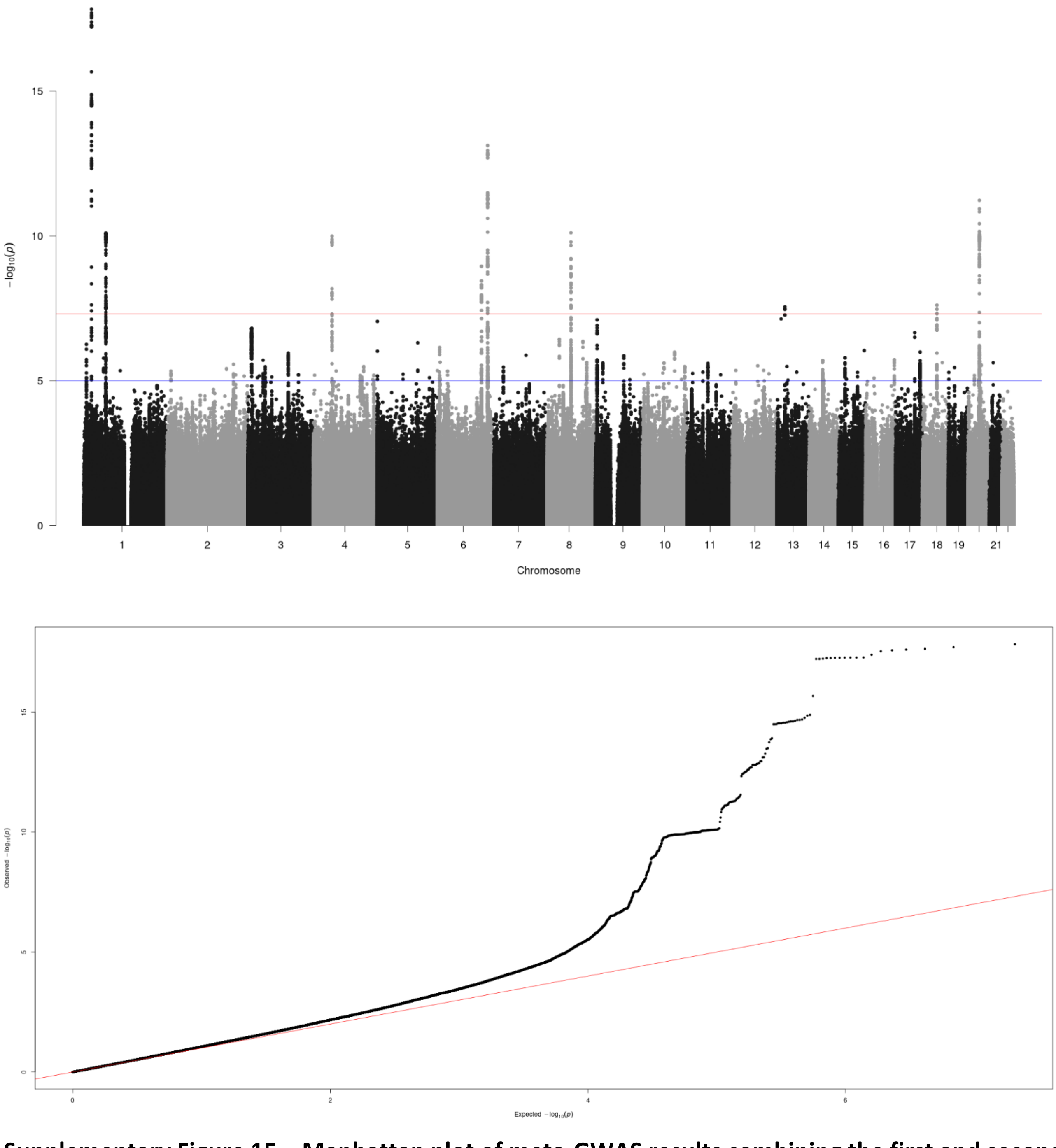

Supplementary Figure 15 – Manhattan plot of meta-GWAS results combining the first and second batch for Femoral head in Females (N=20,536). The Manhattan plot (top) shows the genomic position of each variant on the x-axis and the GWAS negative log10-transformed P-value on the y-axis. Independent lead variants of each locus are annotated by a diamond. The horizontal red line indicates the genome-wide significance threshold that corresponds to a P-value of 5×10^{-8}, while the horizontal blue indicates the suggestive threshold of 1×10^{-5}. The QQ plot (bottom) shows the observed and expected P-value distribution of genetic variants in the meta-GWAS for Femoral head in Females.

SUPPLEMENTARY FIGURE 16

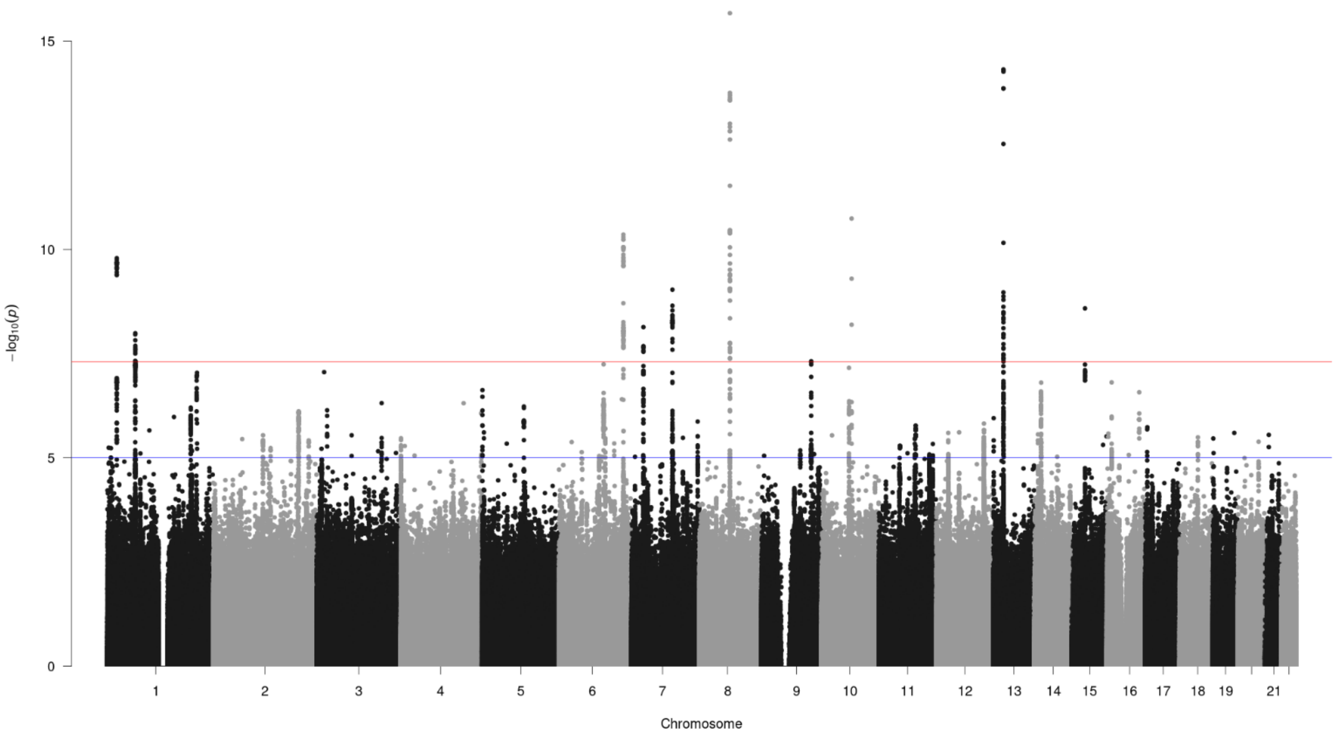

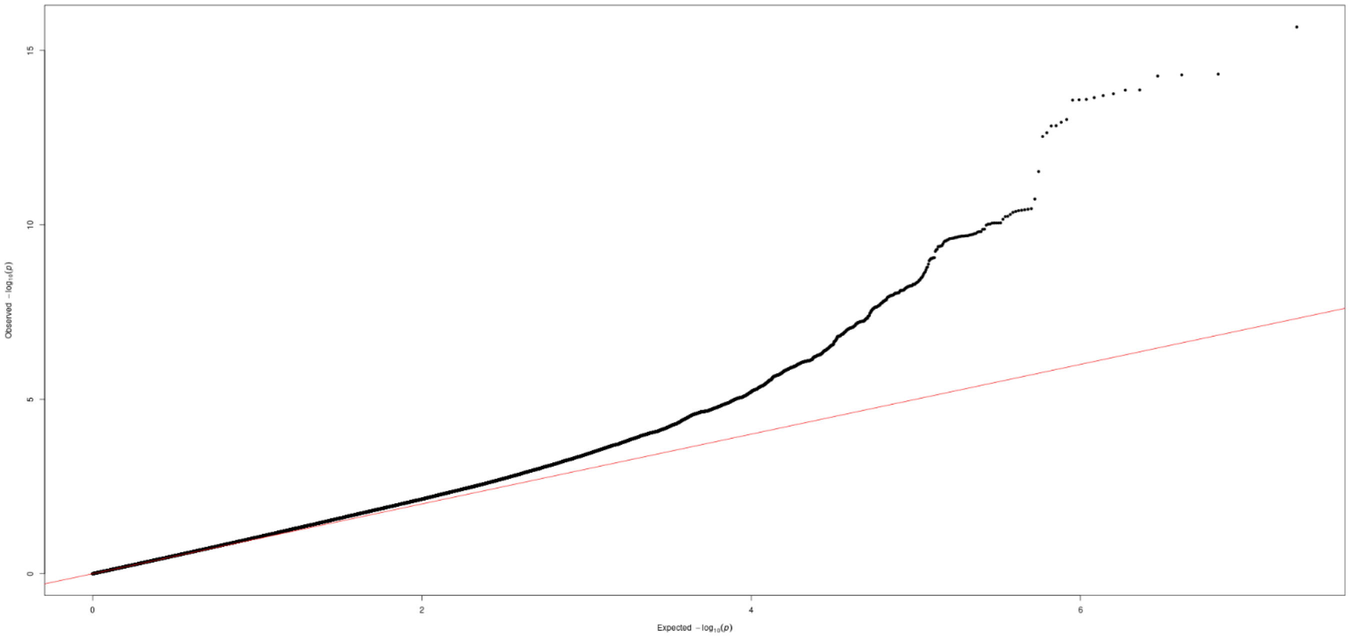

Supplementary Figure 16 – Manhattan plot of meta-GWAS results combining the first and second batch for Total hip in Males (N=17,504). The Manhattan plot (top) shows the genomic position of each variant on the x-axis and the GWAS negative log10-transformed P-value on the y-axis. Independent lead variants of each locus are annotated by a diamond. The horizontal red line indicates the genome-wide significance threshold that corresponds to a P-value of 5×10^{-8}, while the horizontal blue indicates the suggestive threshold of 1×10^{-5}. The QQ plot (bottom) shows the observed and expected P-value distribution of genetic variants in the meta-GWAS for Total hip in Males.

SUPPLEMENTARY FIGURE 17

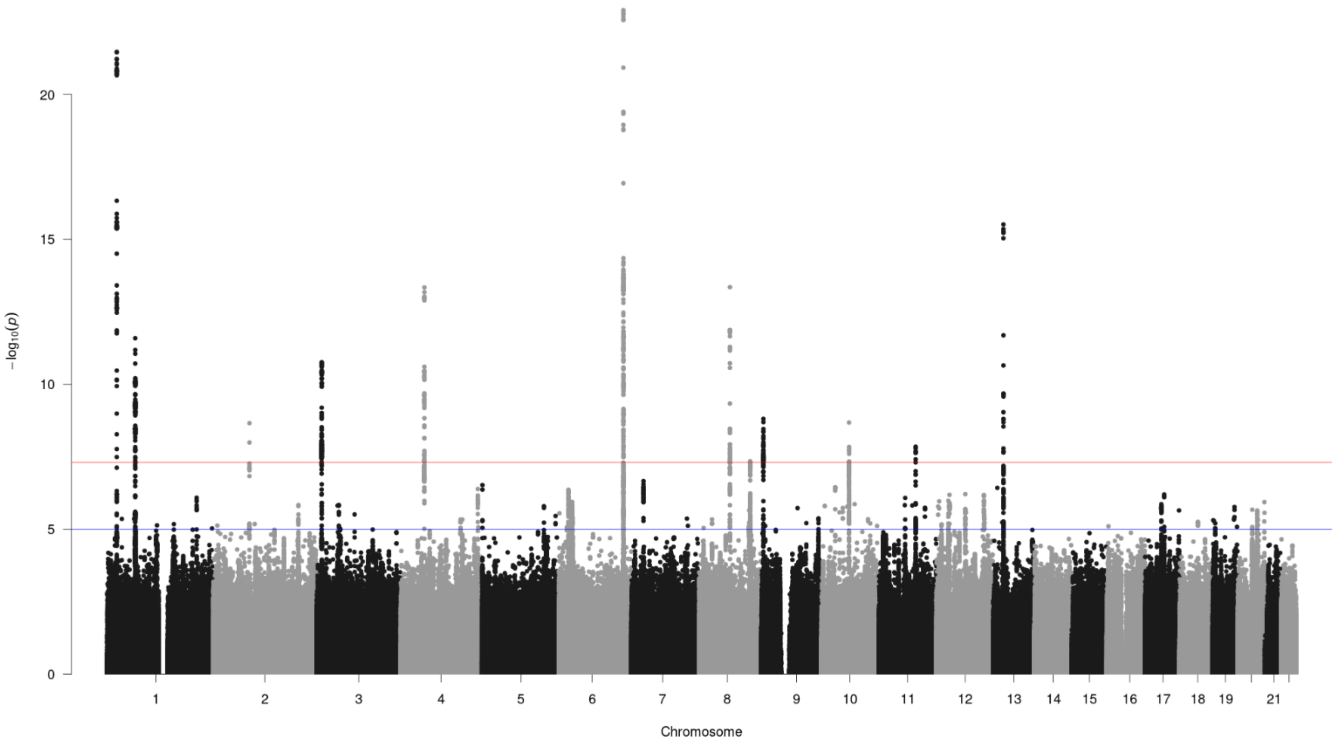

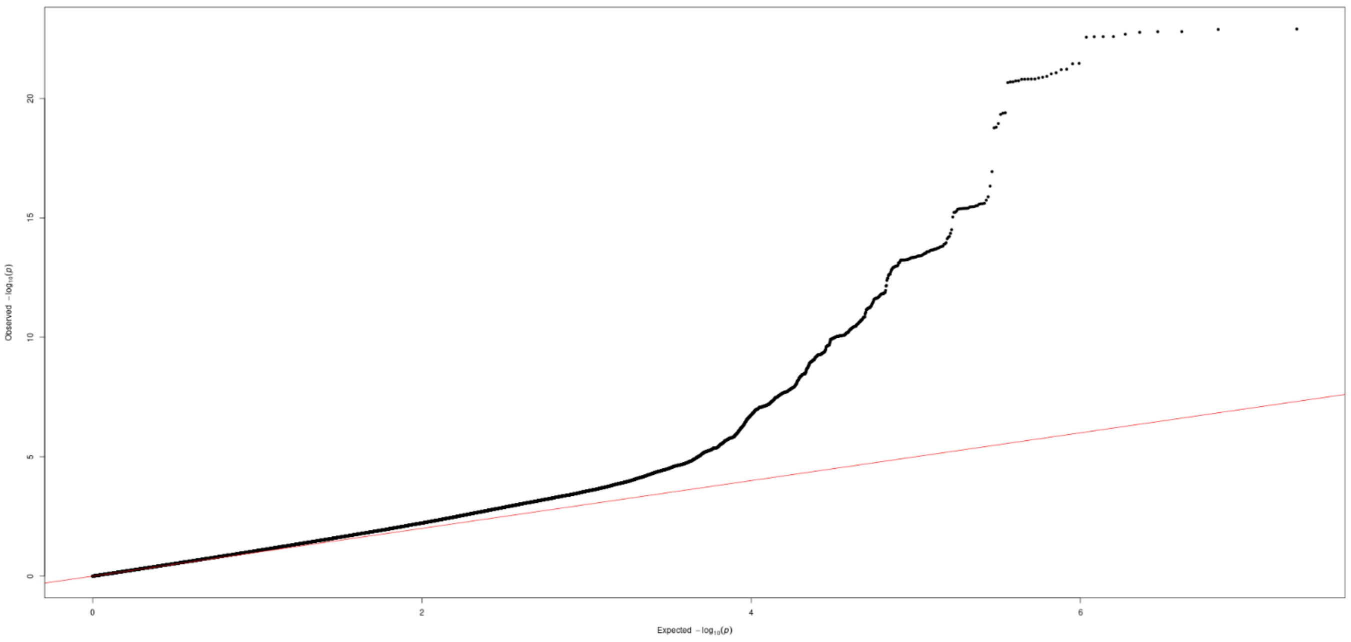

Supplementary Figure 17 – Manhattan plot of meta-GWAS results combining the first and second batch for Total hip in Females (N=20,890). The Manhattan plot (top) shows the genomic position of each variant on the x-axis and the GWAS negative log10-transformed P-value on the y-axis. Independent lead variants of each locus are annotated by a diamond. The horizontal red line indicates the genome-wide significance threshold that corresponds to a P-value of 5×10^{-8}, while the horizontal blue indicates the suggestive threshold of 1×10^{-5}. The QQ plot (bottom) shows the observed and expected P-value distribution of genetic variants in the meta-GWAS for Total hip in Female

SUPPLEMENTARY FIGURE 18

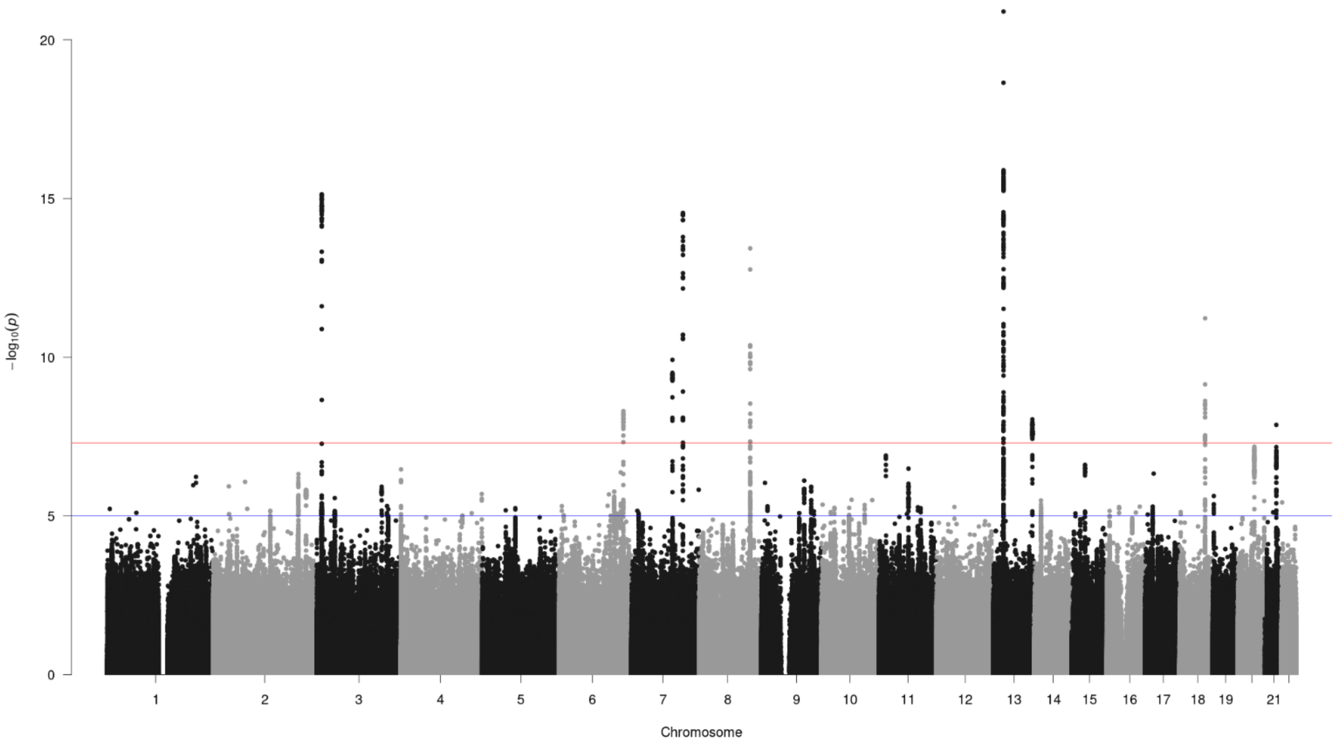

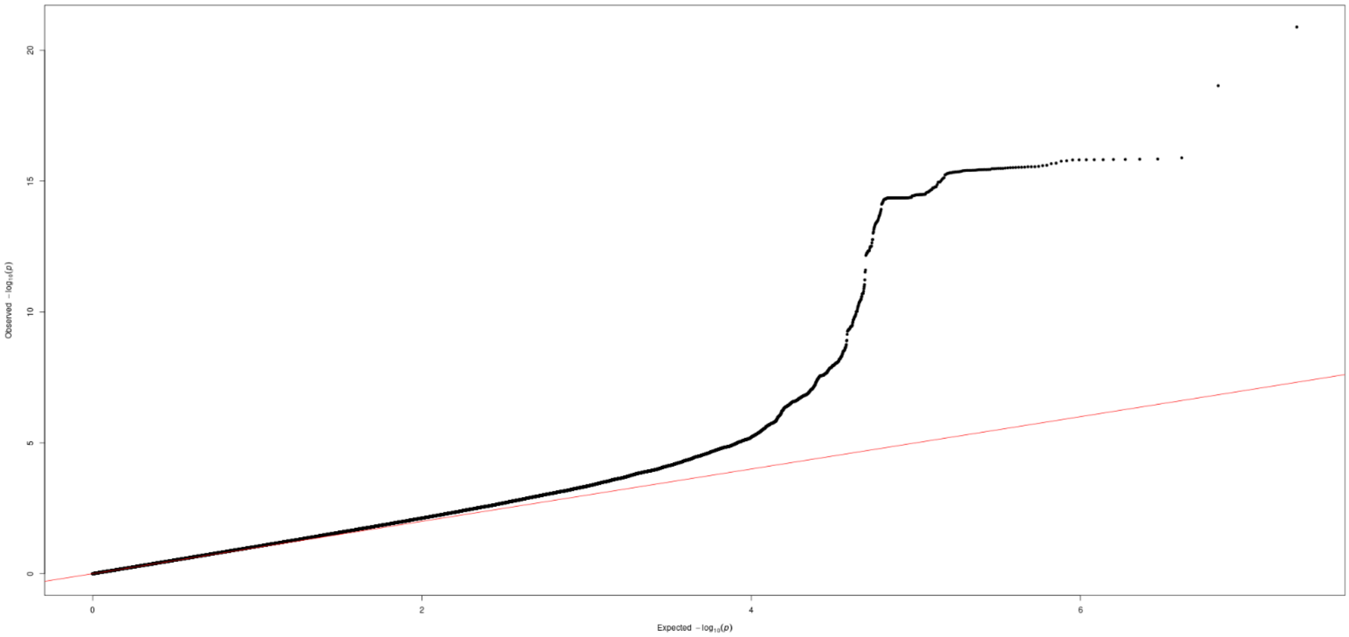

Supplementary Figure 18 – Manhattan plot of meta-GWAS results combining the first and second batch for Diaphysis in Males (N=16,937). The Manhattan plot (top) shows the genomic position of each variant on the x-axis and the GWAS negative log10-transformed P-value on the y-axis. Independent lead variants of each locus are annotated by a diamond. The horizontal red line indicates the genome-wide significance threshold that corresponds to a P-value of 5×10^{-8}, while the horizontal blue indicates the suggestive threshold of 1×10^{-5}. The QQ plot (bottom) shows the observed and expected P-value distribution of genetic variants in the meta-GWAS for Diaphysis in Males.

SUPPLEMENTARY FIGURE 19

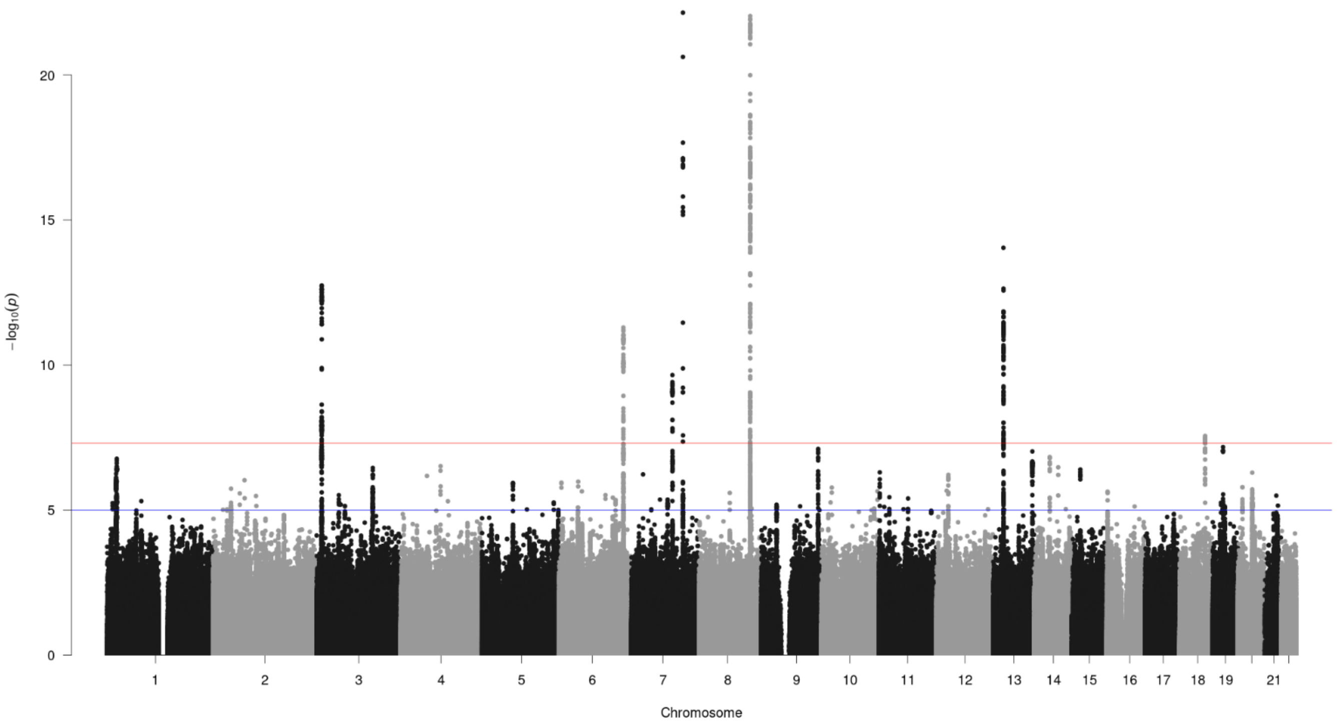

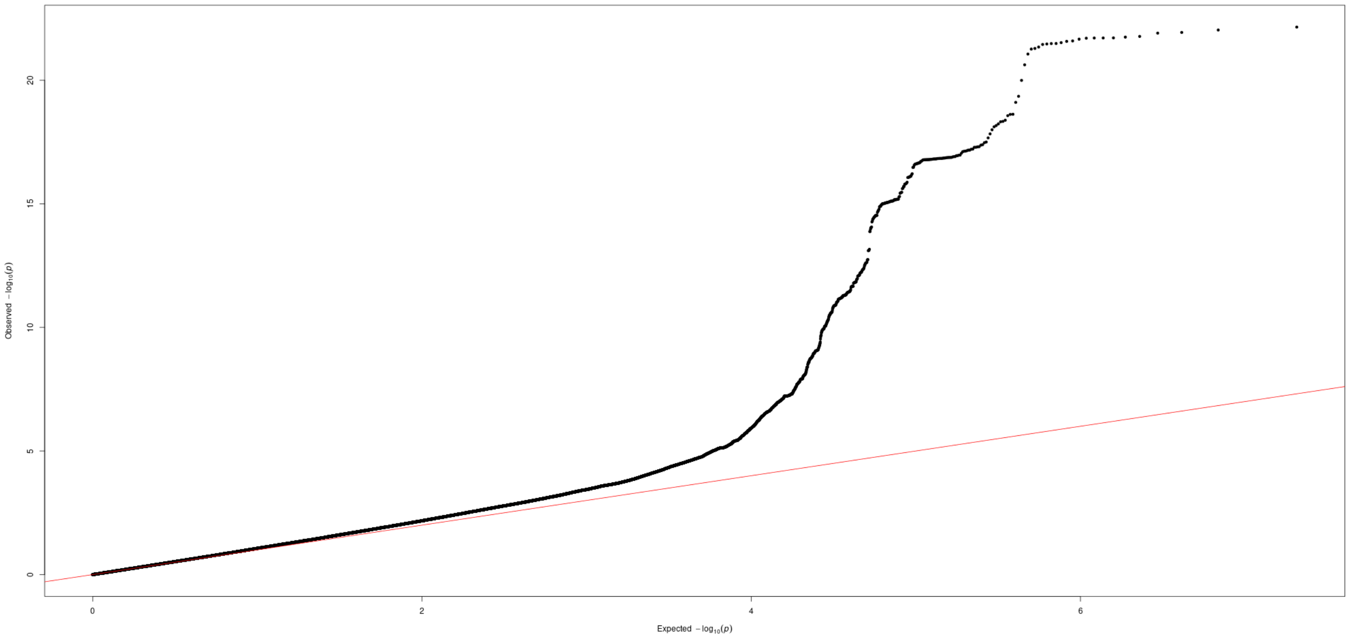

Supplementary Figure 19 – Manhattan plot of meta-GWAS results combining the first and second batch for Diaphysis in Females (N=20,576). The Manhattan plot (top) shows the genomic position of each variant on the x-axis and the GWAS negative log10-transformed P-value on the y-axis. Independent lead variants of each locus are annotated by a diamond. The horizontal red line indicates the genome-wide significance threshold that corresponds to a P-value of 5×10^{-8}, while the horizontal blue indicates the suggestive threshold of 1×10^{-5}. The QQ plot (bottom) shows the observed and expected P-value distribution of genetic variants in the meta-GWAS for Diaphysis in Female.

SUPPLEMENTARY FIGURE 20

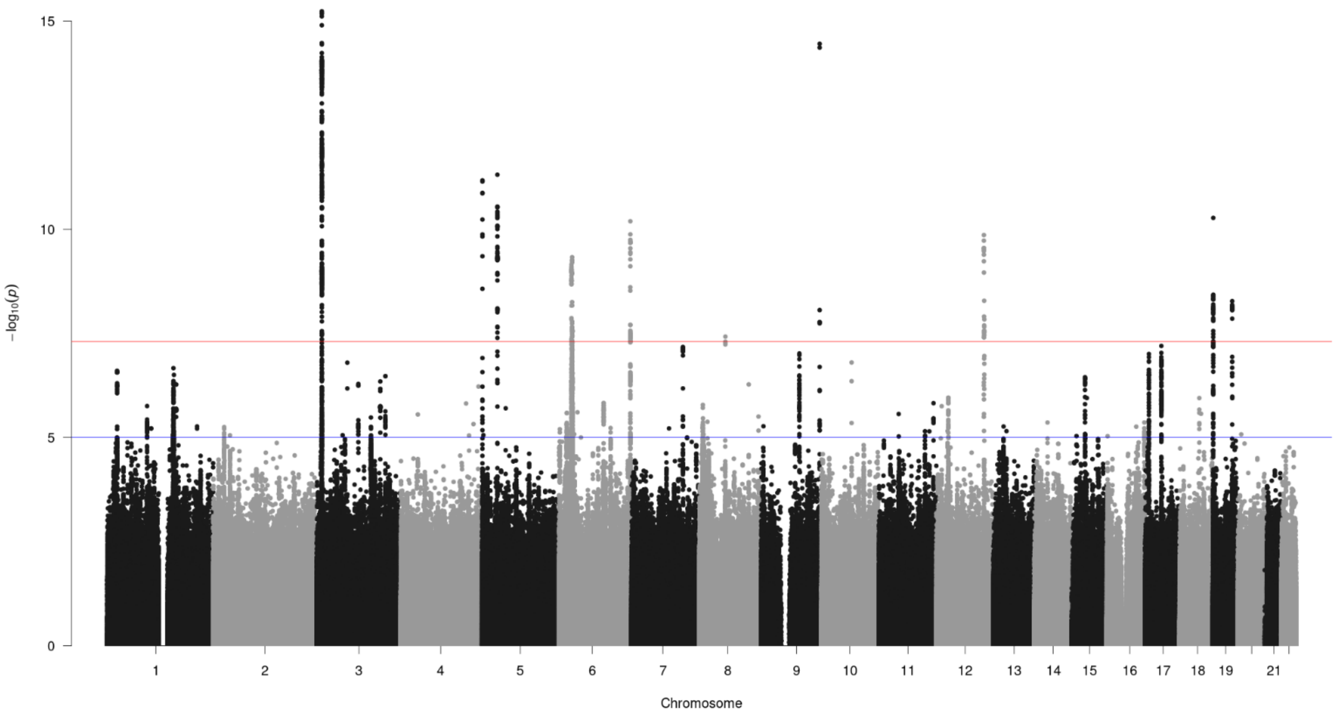

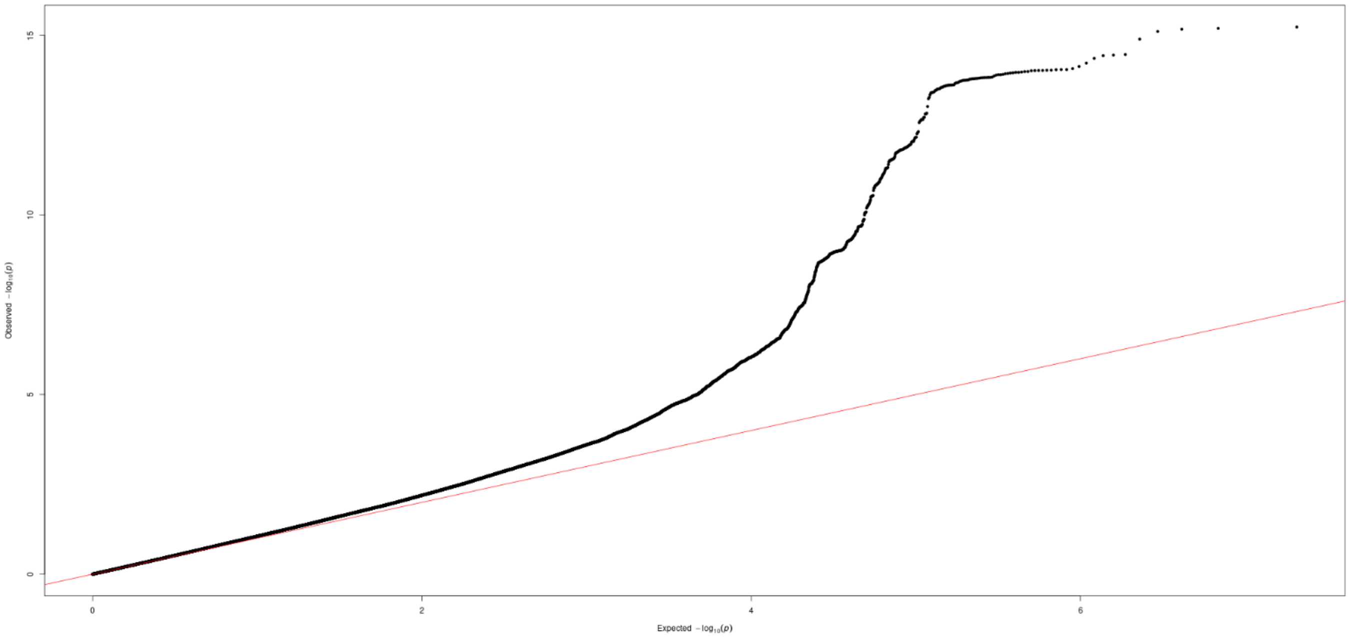

Supplementary Figure 20 – Manhattan plot of meta-GWAS results combining the first and second batch for Spine in Males (N=20,093). The Manhattan plot (top) shows the genomic position of each variant on the x-axis and the GWAS negative log10-transformed P-value on the y-axis. Independent lead variants of each locus are annotated by a diamond. The horizontal red line indicates the genome-wide significance threshold that corresponds to a P-value of 5×10^{-8}, while the horizontal blue indicates the suggestive threshold of 1×10^{-5}. The QQ plot (bottom) shows the observed and expected P-value distribution of genetic variants in the meta-GWAS for Spine in Male.

SUPPLEMENTARY FIGURE 21

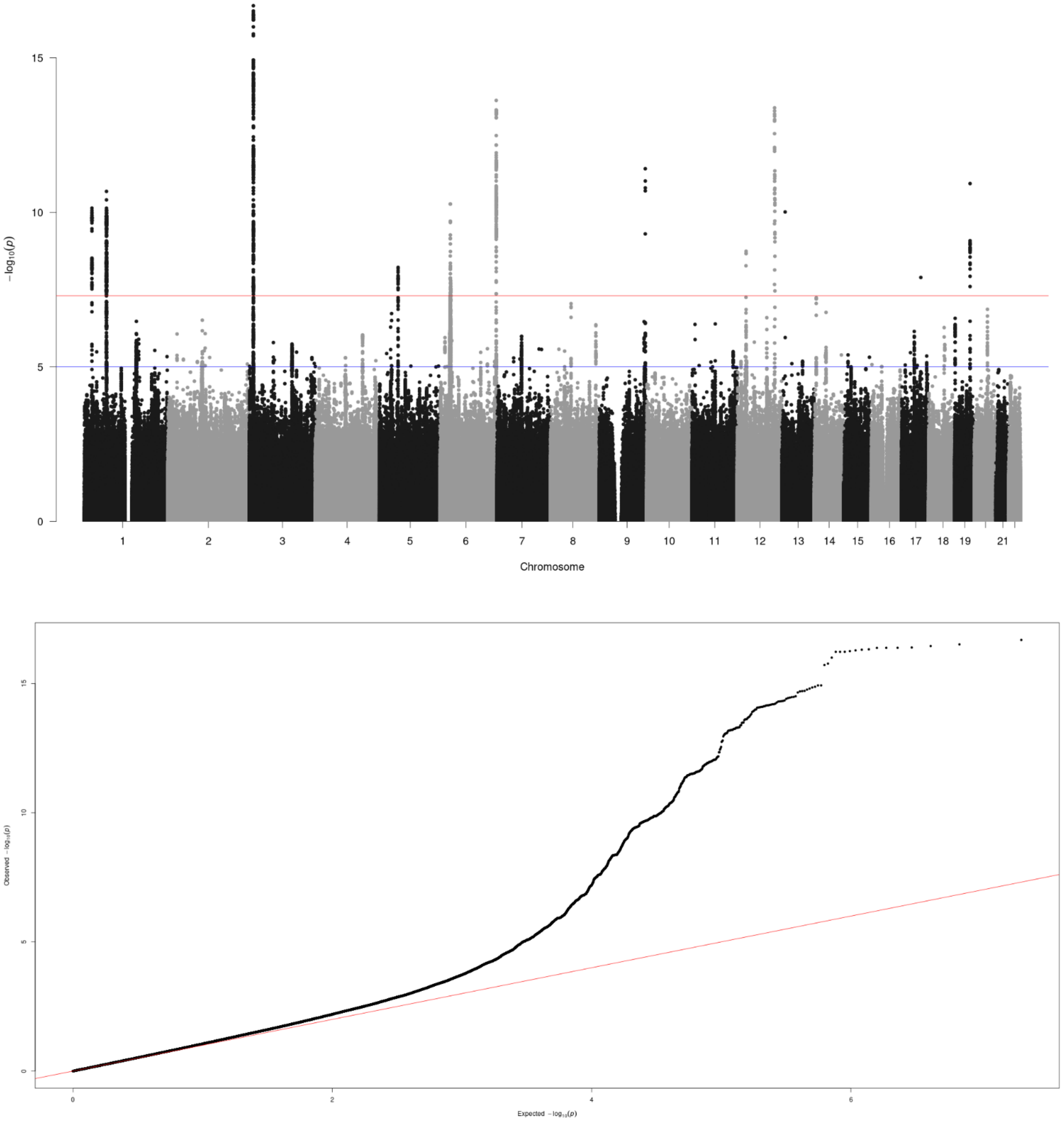

Supplementary Figure 21 – Manhattan plot and QQ plot of the GWAS results for meta-GWAS results combining the first and second batch for Spine in Females (N=21,111). The Manhattan plot (top) shows the genomic position of each variant on the x-axis and the GWAS negative log10-transformed P-value on the y-axis. Independent lead variants of each locus are annotated by a diamond. The horizontal red line indicates the genome-wide significance threshold that corresponds to a P-value of 5×10^{-8}, while the horizontal blue indicates the suggestive threshold of 1×10^{-5}. The QQ plot (bottom) shows the observed and expected P-value distribution of genetic variants in the meta-GWAS for Spine in Females.

SUPPLEMENTARY FIGURE 22

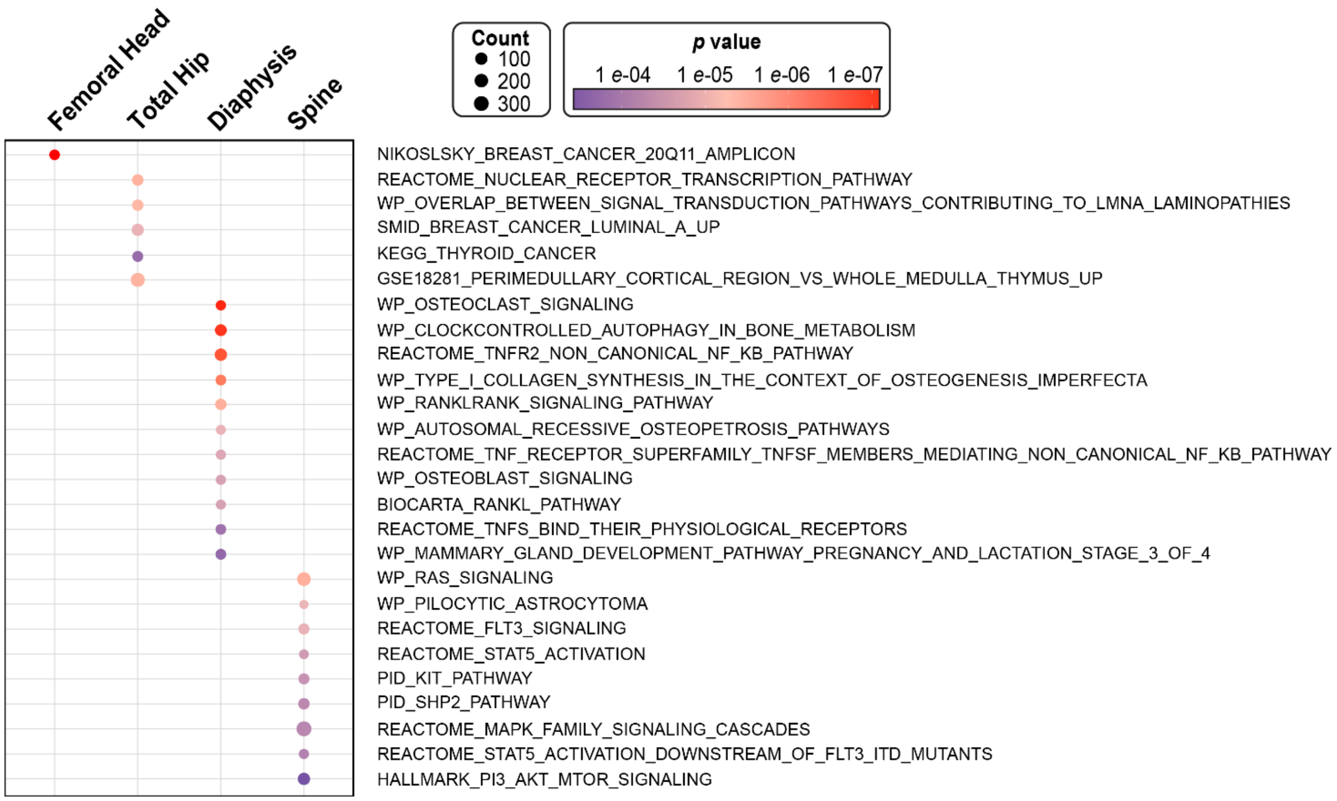

Supplementary Figure 22 – MAGMA gene-set analysis for meta-GWAS in females from the white unrelated population. Bubble plot for significant gene-sets for Meta-GWAS in females after Bonferroni correction showing the region on the x-axis, size and colors of the circles represent the size of the gene-set.

SUPPLEMENTARY FIGURE 23

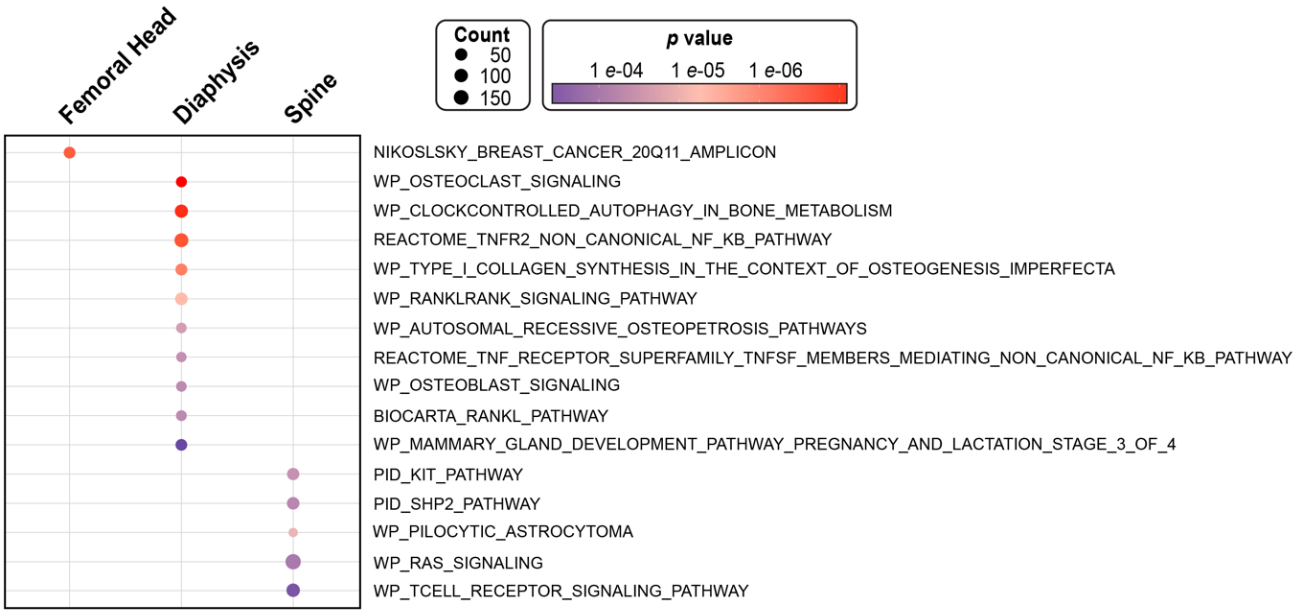

Supplementary Figure 23 – MAGMA gene-set analysis for meta-GWAS in males from the white unrelated population. Bubble plot for significant gene-sets for Meta-GWAS in males after Bonferroni correction showing the region on the x-axis, size and colors of the circles represent the size of the gene-set.

SUPPLEMENTARY FIGURE 24

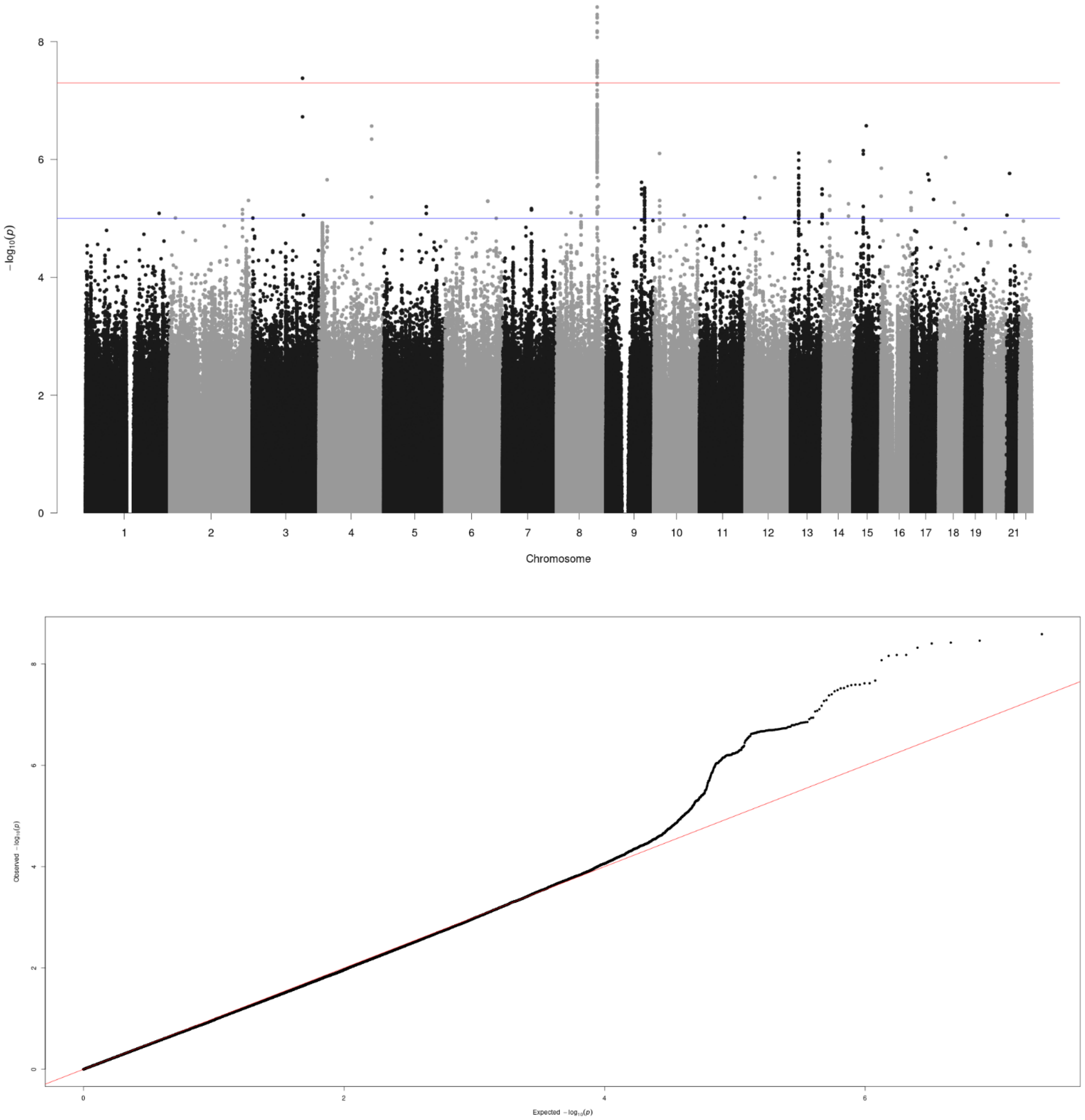

Supplementary Figure 24 – Manhattan plot and QQ plot of the GWAS results for the two batches combined in non-white for femoral head (N=5933). The Manhattan plot (top) shows the genomic position of each variant on the x-axis and the GWAS negative log10-transformed P-value on the y-axis. Independent lead variants of each locus are annotated by a diamond. The horizontal red line indicates the genome-wide significance threshold that corresponds to a P-value of 5×10^{-8}, while the horizontal blue indicates the suggestive threshold of 1×10^{-5}. The QQ plot (bottom) shows the observed and expected P-value distribution of genetic variants.

SUPPLEMENTARY FIGURE 25

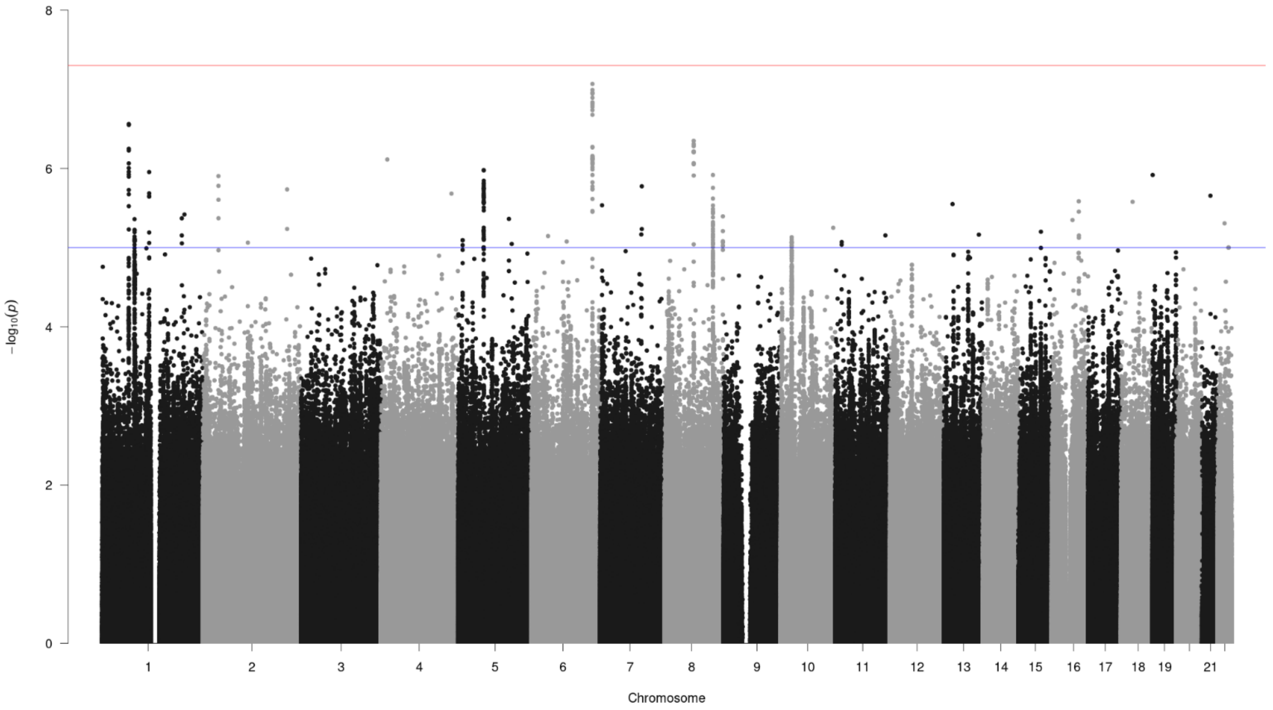

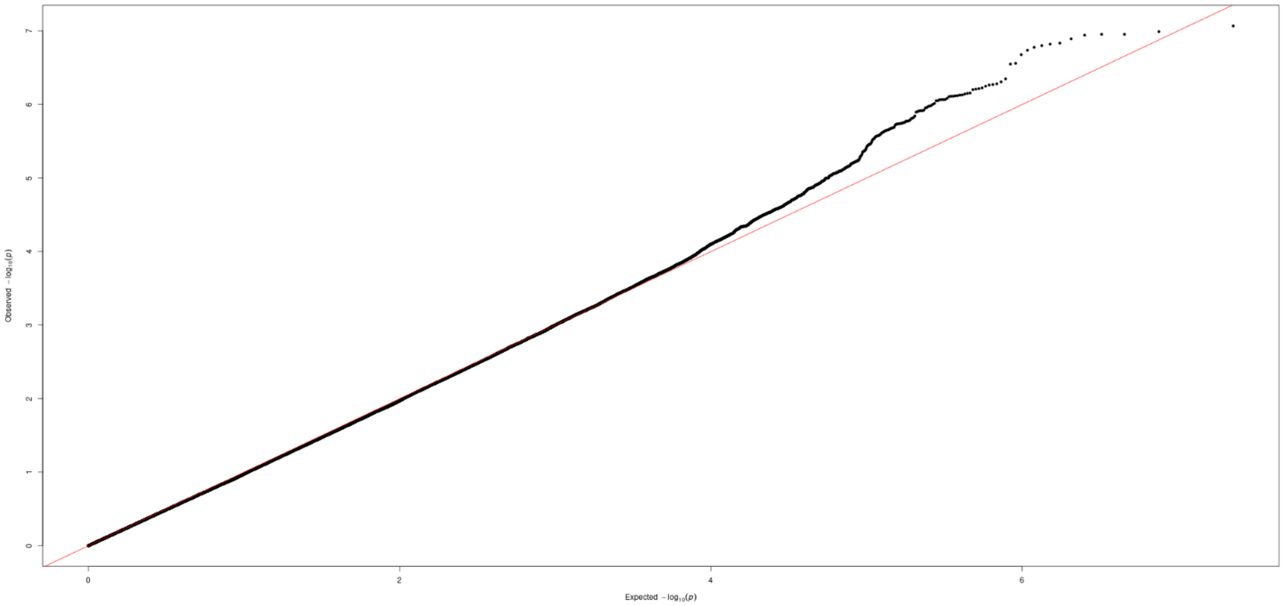

Supplementary Figure 25 – Manhattan plot and QQ plot of the GWAS results for the two batches combined in non-white for total hip (N=6055). The Manhattan plot (top) shows the genomic position of each variant on the x-axis and the GWAS negative log10-transformed P-value on the y-axis. Independent lead variants of each locus are annotated by a diamond. The horizontal red line indicates the genome-wide significance threshold that corresponds to a P-value of 5×10^{-8}, while the horizontal blue indicates the suggestive threshold of 1×10^{-5}. The QQ plot (bottom) shows the observed and expected P-value distribution of genetic variants.

SUPPLEMENTARY FIGURE 26

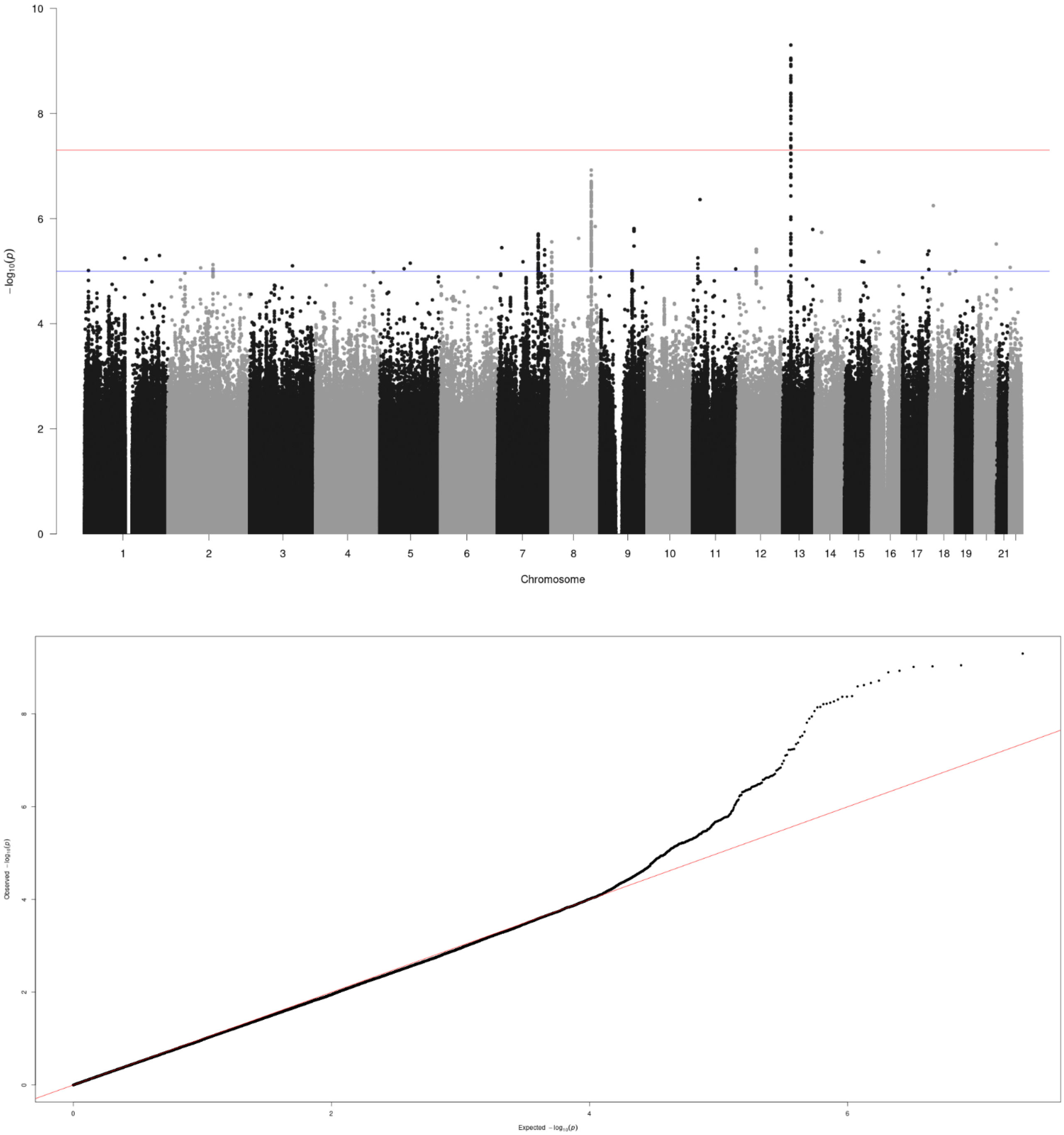

Supplementary Figure 26 – Manhattan plot and QQ plot of the GWAS results for the two batches combined in non-white for diaphysis (N=5844). The Manhattan plot (top) shows the genomic position of each variant on the x-axis and the GWAS negative log10-transformed P-value on the y-axis. Independent lead variants of each locus are annotated by a diamond. The horizontal red line indicates the genome-wide significance threshold that corresponds to a P-value of 5×10^{-8}, while the horizontal blue indicates the suggestive threshold of 1×10^{-5}. The QQ plot (bottom) shows the observed and expected P-value distribution of genetic variants.

SUPPLEMENTARY FIGURE 27

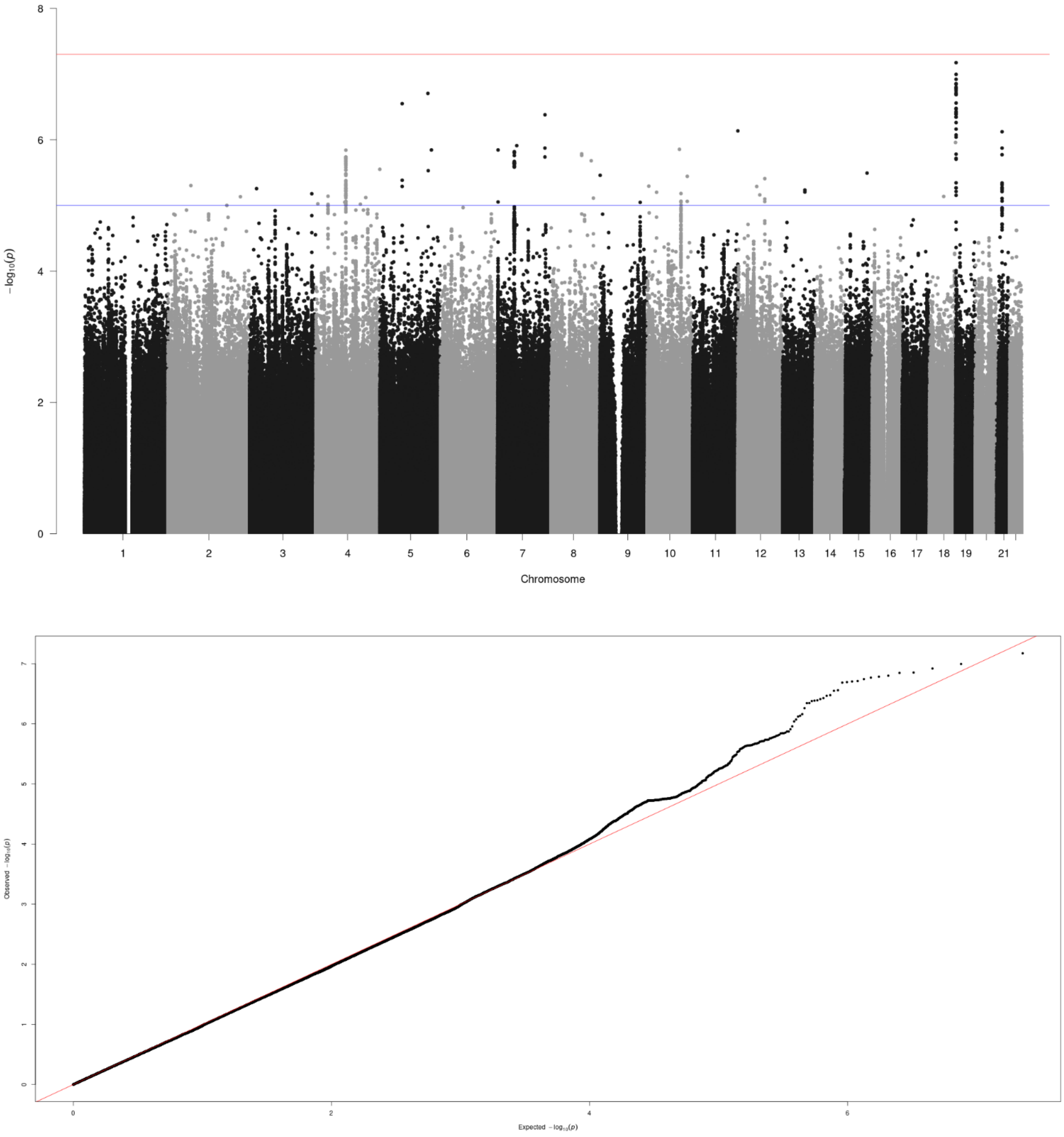

Supplementary Figure 27 – Manhattan plot and QQ plot of the GWAS results for the two batches combined in non-white for spine (N=6367). The Manhattan plot (top) shows the genomic position of each variant on the x-axis and the GWAS negative log10-transformed P-value on the y-axis. Independent lead variants of each locus are annotated by a diamond. The horizontal red line indicates the genome-wide significance threshold that corresponds to a P-value of 5×10^{-8}, while the horizontal blue indicates the suggestive threshold of 1×10^{-5}. The QQ plot (bottom) shows the observed and expected P-value distribution of genetic variants.

SUPPLEMENTARY FIGURE 28

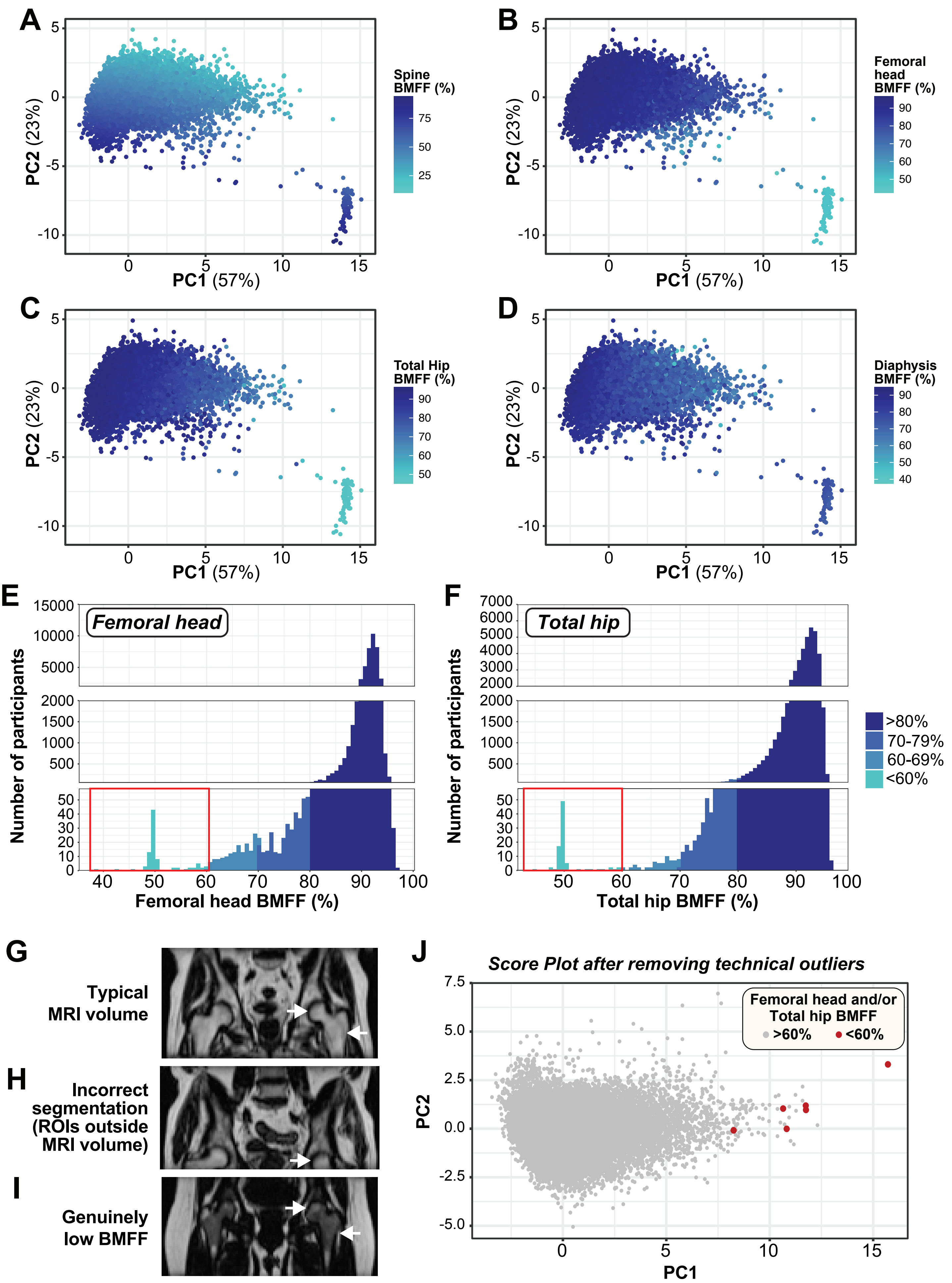

Supplementary Figure 28 – Analysis of technical outliers from deep learning segmentation and BMFF analysis of 46,717 UKBB participants. (A-D) Score plots of principal components 1 and 2 (PC1 and PC2, respectively) from principal component analysis (PCA) of BMFF values for the four regions, calculated across the full cohort (batch 1 and batch 2 combined; n=46,717). Note that n differs to that used in Figures 2-4 because the PCA used a merged dataframe to ensure the same number of participants for each site: n decreased from 47,571 to 46,717 for spine BMFF and increased to 46,717 for each femoral region (with missing femoral BMFF values imputed using random forest imputation). PC1 explains 57% of the variance and PC2 23% of the variance, as indicated in the axis titles. Plots are colored according to BMFF % for the Spine (A), Femoral Head (B), Total Hip (C), or Diaphysis (D). **(E-F)** Histograms of BMFF % for the Femoral head (E) and Total hip (F) for the full cohort (n=46,717). Bars are colored according to stratified ranges of BMFF, as indicated. The distinct group of participants around 50% BMFF highlight the technical outliers in the full cohort. **(G-I)** Representative coronal MRI volumes for the Femoral Head and Total Hip regions for a technical outlier (G), a biological outlier (H), and a typical volume (I); in (H-I), arrows indicate the expected locations of the Femoral Head and Total Hip. In (G), these regions fall below the imaging volume, resulting in incorrect segmentation and inaccurate BMFF measurement. In (H), the regions are correctly segmented but have genuinely low BMFF %, indicating a biological outlier. **(J)** Score plot of PC1 vs PC2 from PCA of the current cohort after excluding technical outliers (n=46,633). The remaining biological outliers, with Femoral Head and/or Total hip BMFF <60%, are indicated by red dots. Please see Methods, "Principal component analysis and MRI image error checking to identify technical outliers from deep learning segmentation", for further details.
