# Supplementary material for: Deep learning and genome-wide association meta-analyses of bone marrow adiposity in the UK Biobank
Source: Nat Commun. 2025 Jan 2;16:99. doi: 10.1038/s41467-024-55422-4 (PMC11697225; doi:10.1038/s41467-024-55422-4)
Supplement: Supplementary file 3 — Description of additional supplementary files [file 41467_2024_55422_MOESM3_ESM.pdf]

Description of additional supplementary files

File Name: Supplementary Data 1
Description: Quality control process for the UK Biobank sample of white unrelated individuals from the first batch

File Name: Supplementary Data 2
Description: Quality control process for the UK Biobank sample of white unrelated individuals from the second batch

File Name: Supplementary Data 3
Description: Summary characteristics of the white unrelated sample from the first batch

File Name: Supplementary Data 4
Description: Summary characteristics of the white unrelated sample from the second batch

File Name: Supplementary Data 5
Description: Quality control process for the UK Biobank sample of non-white unrelated individuals

File Name: Supplementary Data 6
Description: Summary characteristics of the non-white unrelated sample

File Name: Supplementary Data 7
Description: Summary of BMFF comparison in white and non-white subgroups

File Name: Supplementary Data 8
Description: Age- and sex-stratified reference ranges for BMFF of each bone region in white and non-white participants

File Name: Supplementary Data 9
Description: Associations between BMFF and age, BMI, or body composition traits in white and non-white participants, with or without controlling for BMD

File Name: Supplementary Data 10
Description: LDSC results for the GWAS of the two batches for white unrelated samples

File Name: Supplementary Data 11
Description: Lead SNPs in meta-GWAS in the sample of white unrelated participants

File Name: Supplementary Data 12
Description: Independent significant SNPs in meta-GWAS in the sample of white unrelated participants

File Name: Supplementary Data 13
Description: Mapped genes in meta-GWAS in the sample of white unrelated participants

File Name: Supplementary Data 14
Description: Lead SNPs in meta-GWAS for the white unrelated sample (without BMI adjustment)

File Name: Supplementary Data 15
Description: Comparison of lead SNPs in meta-GWAS for the white unrelated sample (with/without BMI adjustment)

File Name: Supplementary Data 16
Description: Independent significant SNPs in meta-GWAS for the white unrelated sample (without BMI adjustment)

File Name: Supplementary Data 17
Description: Comparison of independent significant SNPs in meta-GWAS for the white unrelated sample (with/without BMI adjustment)

File Name: Supplementary Data 18
Description: Mapped genes in meta-GWAS for the white unrelated sample (without BMI adjustment)

File Name: Supplementary Data 19
Description: Lead SNPs in meta-GWAS for the white unrelated sample ($r2<0.3$, $p<5e-08$)

File Name: Supplementary Data 20
Description: Independent significant SNPs in meta-GWAS for the white unrelated sample ($r2<0.3$, $p<5e-08$)

File Name: Supplementary Data 21
Description: Lead SNPs in meta-GWAS for the white unrelated sample ($r2<0.3$, $p<1e-08$)

File Name: Supplementary Data 22
Description: Independent significant SNPs in meta-GWAS for the white unrelated sample ($r2<0.3$, $p<1e-08$)

File Name: Supplementary Data 23
Description: Summary of lead and independent significant SNPs using two thresholds in meta-GWAS for the white population

File Name: Supplementary Data 24
Description: MAGMA tissue expression analysis in meta-GWAS in white unrelated sample (54 tissues in GTEx database v8)

File Name: Supplementary Data 25
Description: MAGMA Cell-type-specific gene set analysis in meta-GWAS in white unrelated sample (mouse-derived bone marrow types)

File Name: Supplementary Data 26
Description: TWAS associations between gene expression and BMFF based on meta-GWAS-white for femoral head (subcutaneous adipose tissue; visceral-omentum adipose tissue; muscle skeleton tissue in GTEx database v8)

File Name: Supplementary Data 27
Description: TWAS associations between gene expression and BMFF based on meta-GWAS-white for total hip (subcutaneous adipose tissue; visceral-omentum adipose tissue; muscle skeleton tissue in GTEx database v8)

File Name: Supplementary Data 28

Description: TWAS associations between gene expression and BMFF based on meta-GWAS-white for diaphysis (subcutaneous adipose tissue; visceral-omentum adipose tissue; muscle skeleton tissue in GTEx database v8)

File Name: Supplementary Data 29
Description: TWAS associations between gene expression and BMFF based on meta-GWAS-white for spine (subcutaneous adipose tissue; visceral-omentum adipose tissue; muscle skeleton tissue in GTEx database v8)

File Name: Supplementary Data 30
Description: Comparison of overlapping significant TWAS associations between gene expression and BMFF based on meta-GWAS-white (subcutaneous adipose tissue; visceral-omentum adipose tissue; muscle skeleton tissue in GTEx database v8)

File Name: Supplementary Data 31
Description: Colocalization of eQTL signals with BMFF meta-GWAS-white signals (subcutaneous adipose tissue; visceral-omentum adipose tissue; muscle skeleton tissue; spleen tissue; Cells_EBV-transformed_lymphocytes tissue in GTEx database v8)

File Name: Supplementary Data 32
Description: Summary of GWAS catalog for meta-GWAS in white unrelated sample for femoral head

File Name: Supplementary Data 33
Description: Summary of GWAS catalog for meta-GWAS in white unrelated sample for total hip

File Name: Supplementary Data 34
Description: Summary of GWAS catalog for meta-GWAS in white unrelated sample for femoral diaphysis

File Name: Supplementary Data 35
Description: Summary of GWAS catalog for meta-GWAS in white unrelated sample for spine

File Name: Supplementary Data 36
Description: Cross-trait LD score regression comparing BMFF and other traits

File Name: Supplementary Data 37
Description: Summary characteristics of the white unrelated sample from the first batch by sex group

File Name: Supplementary Data 38
Description: Summary characteristics of the white unrelated sample from the second batch by sex group

File Name: Supplementary Data 39
Description: Lead SNPs in meta-GWAS in the sample of white unrelated participants by sex group

File Name: Supplementary Data 40
Description: Independent significant SNPs in meta-GWAS in the sample of white unrelated participants by sex group

File Name: Supplementary Data 41

Description: Mapped genes in meta-GWAS in the sample of white unrelated participants by sex group

File Name: Supplementary Data 42
Description: Comparison of overlapping mapped genes in meta-GWAS in white unrelated sample by sex group

File Name: Supplementary Data 43
Description: Lead SNPs in meta-GWAS for the white unrelated sample by sex group (without BMI adjustment)

File Name: Supplementary Data 44
Description: Comparison of lead SNPs in meta-GWAS for the white unrelated sample by sex group (with/without BMI adjustment)

File Name: Supplementary Data 45
Description: Independent significant SNPs in meta-GWAS for the white unrelated sample by sex group (without BMI adjustment)

File Name: Supplementary Data 46
Description: Comparison of independent significant SNPs in meta-GWAS for the white unrelated sample by sex group (with/without BMI adjustment)

File Name: Supplementary Data 47
Description: Mapped genes in meta-GWAS for the white unrelated sample by sex group (without BMI adjustment)

File Name: Supplementary Data 48
Description: Lead SNPs in meta-GWAS for the white unrelated sample by sex group (r2<0.3, p<5e-08)

File Name: Supplementary Data 49
Description: Independent significant SNPs in meta-GWAS for the white unrelated sample by sex group (r2<0.3, p<5e-08)

File Name: Supplementary Data 50
Description: Lead SNPs in meta-GWAS for the white unrelated sample by sex group (r2<0.3, p<1e-08)

File Name: Supplementary Data 51
Description: Independent significant SNPs in meta-GWAS for the white unrelated sample by sex group (r2<0.3, p<1e-08)

File Name: Supplementary Data 52
Description: Summary of lead and independent significant SNPs using two thresholds in meta-GWAS for the white population by sex group

File Name: Supplementary Data 53
Description: Sex x Genotype interaction of lead SNPs in meta-GWAS for the white unrelated sample

File Name: Supplementary Data 54

Description: MAGMA tissue expression analysis in meta-GWAS in white unrelated sample by sex group (54 tissues in GTEx database v8)

File Name: Supplementary Data 55
Description: MAGMA Cell-type-specific gene set analysis in meta-GWAS in white unrelated sample by sex group (mouse-derived bone marrow types)

File Name: Supplementary Data 56
Description: Summary of GWAS catalog for meta-GWAS in white unrelated sample by sex group for femoral head

File Name: Supplementary Data 57
Description: Summary of GWAS catalog for meta-GWAS in white unrelated sample by sex group for total hip

File Name: Supplementary Data 58
Description: Summary of GWAS catalog for meta-GWAS in white unrelated sample by sex group for femoral diaphysis

File Name: Supplementary Data 59
Description: Summary of GWAS catalog for meta-GWAS in white unrelated sample by sex group for spine

File Name: Supplementary Data 60
Description: Independent significant SNPs and mapped genes in GWAS in non-white unrelated sample

File Name: Supplementary Data 61
Description: Lead SNPs in GWAS for non-white ethnic subgroups

File Name: Supplementary Data 62
Description: Independent significant SNPs in GWAS for non-white ethnic subgroups

File Name: Supplementary Data 63
Description: Mapped genes in GWAS for non-white ethnic subgroups

File Name: Supplementary Data 64
Description: GWAS power for non-white ethnic subgroups

File Name: Supplementary Data 65
Description: Lead SNPs in meta-GWAS in non-white and white unrelated sample

File Name: Supplementary Data 66
Description: Independent significant SNPs in meta-GWAS in non-white and white unrelated sample

File Name: Supplementary Data 67
Description: Mapped genes in meta-GWAS in non-white and white unrelated sample

File Name: Supplementary Data 68
Description: Comparison of overlapping mapped genes in meta-GWAS in non-white and white unrelated sample

File Name: Supplementary Data 69
Description: MAGMA tissue expression analysis in meta-GWAS in non-white and white unrelated sample (54 tissues in GTEx database v8)

File Name: Supplementary Data 70
Description: MAGMA Cell-type-specific gene set analysis in meta-GWAS in non-white and white unrelated sample (mouse-derived bone marrow types)

File Name: Supplementary Data 71
Description: Summary of GWAS catalog for meta-GWAS in non-white and white unrelated sample for femoral head

File Name: Supplementary Data 72
Description: Summary of GWAS catalog for meta-GWAS in non-white and white unrelated sample for total hip

File Name: Supplementary Data 73
Description: Summary of GWAS catalog for meta-GWAS in non-white and white unrelated sample for femoral diaphysis

File Name: Supplementary Data 74
Description: Summary of GWAS catalog for meta-GWAS in non-white and white unrelated sample for spine
