# Supplementary material for: Deep learning and genome-wide association meta-analyses of bone marrow adiposity in the UK Biobank
Source: Nat Commun. 2025 Jan 2;16:99. doi: 10.1038/s41467-024-55422-4 (PMC11697225; doi:10.1038/s41467-024-55422-4)
Supplement: Supplementary file 78 — Reporting Summary [file 41467_2024_55422_MOESM78_ESM.pdf]

nature portfolio

Corresponding author(s): William P Cawthorn and Evropi Theodoratou

Last updated by author(s): 17 November, 2024

Reporting Summary

Nature Portfolio wishes to improve the reproducibility of the work that we publish. This form provides structure for consistency and transparency in reporting. For further information on Nature Portfolio policies, see our Editorial Policies and the Editorial Policy Checklist.

Statistics

For all statistical analyses, confirm that the following items are present in the figure legend, table legend, main text, or Methods section.

n/a	Confirmed	
☐	☒	The exact sample size (*n*) for each experimental group/condition, given as a discrete number and unit of measurement
☐	☒	A statement on whether measurements were taken from distinct samples or whether the same sample was measured repeatedly
☐	☒	The statistical test(s) used AND whether they are one- or two-sided<br>*Only common tests should be described solely by name; describe more complex techniques in the Methods section.*
☐	☒	A description of all covariates tested
☐	☒	A description of any assumptions or corrections, such as tests of normality and adjustment for multiple comparisons
☐	☒	A full description of the statistical parameters including central tendency (e.g. means) or other basic estimates (e.g. regression coefficient) AND variation (e.g. standard deviation) or associated estimates of uncertainty (e.g. confidence intervals)
☐	☒	For null hypothesis testing, the test statistic (e.g. *F*, *t*, *r*) with confidence intervals, effect sizes, degrees of freedom and *P* value noted<br>*Give P values as exact values whenever suitable.*
☒	☐	For Bayesian analysis, information on the choice of priors and Markov chain Monte Carlo settings
☒	☐	For hierarchical and complex designs, identification of the appropriate level for tests and full reporting of outcomes
☐	☒	Estimates of effect sizes (e.g. Cohen's *d*, Pearson's *r*), indicating how they were calculated

Our web collection on statistics for biologists contains articles on many of the points above.

Software and code

Policy information about availability of computer code

Data collection	Data collection was done separately by UK Biobank and not directly as part of this study. No software was used for data collection.
Data analysis	Our previous manuscript (https://doi.org/10.1016/j.csbj.2023.12.029) describes the methods for analysis of bone marrow fat fraction (BMFF) from UK Biobank MRI data. This includes:<br>- Deep learning for segmentation of bone marrow volumes in the spine, femoral head, total hip, and femoral diaphysis (code: Python). This code is available at DOI: 10.5281/zenodo.13959673 and at https://github.com/chengjiawang/OPTIMAT_NET/tree/iniRelease.<br>- Code for sorting UK Biobank MRI data (prior to segmentation) and for fat fraction mapping (code: Matlab). This code is available at DOI: 10.5281/zenodo.13961316 and at https://github.com/WillCawthorn/OPTIMAT.<br><br>Availability of code relating to our GWAS analyses is as follows:<br>- GWAS: We performed GWAS analyses to investigate associations between imputed genotypes and BMFF. These analyses were adjusted for age at imaging visit, sex, BMI at imaging visit, genotyping batch, and population structure of the first 40 principal components (PCs 1-40). These analyses were done by regressing rank-transformed BMFF residuals against HRC imputed genotype dosages using RegScan v0.5 (https://genomics.ut.ee/en/tools).<br>- Meta-GWAS-white: We conducted meta-analyses of GWASes in the first and second BMFF batches for white population under IVW fixed effects models for the four bone regions respectively, using META v1.7 (https://mathgen.stats.ox.ac.uk/genetics_software/meta/meta.html).<br>- Multi-ancestry meta-GWAS: We performed multi-ancestry meta-analyses of white and non-white populations for each bone region using the<br>- Meta-Regression of Multi-Ethnic Genetic Association (MR-MEGA v0.20). (https://genomics.ut.ee/en/tools)<br>- GWAS power calculation: We performed GWAS power calculation using GCTA package (https://yanglab.westlake.edu.cn/software/gcta/#GREMLpowercalculator).<br>- Sex x Genotype interaction: We analyzed the sex x genotype interaction using the formula to calculate t-sex and P-sex, as developed by

Winkler et al (https://doi.org/10.1371/journal.pgen.1005378).
- LDSC: We used linkage disequilibrium score regression to estimate genomic inflation, SNP-based heritability and genetic correlations (rg). (LDSC: https://github.com/bulik/ldsc)
- TWAS: We conducted TWAS analysis using FUSION, by integrating gene expression prediction models generated from subcutaneous adipose tissue, visceral-omentum adipose tissue and skeletal muscle tissue (GTEx v8) with meta-GWAS for BMFF in the white population (http://gusevlab.org/projects/fusion/). The TWAS Z-score plot was generated using a TWAS-plotter function (https://github.com/opain/TWAS-plotter).
- Colocalization: We performed colocalization to determine whether the same genetic variant was responsible for both an eQTL effect (mesodermal and lymphoid tissues from GTEx v8) and a meta-GWAS signal (BMFF trait). Colocalization was performed using 'coloc.abf' function from Coloc R package.
- FUMA: We used FUMA v1.5.2 for functional annotation of the Meta-GWAS-white and MR-MEGA summary statistics. We performed MAGMA gene-set analysis, tissue expression analysis and cell-type-specific gene expression analysis using FUMA (http://fuma.ctglab.nl).

For manuscripts utilizing custom algorithms or software that are central to the research but not yet described in published literature, software must be made available to editors and reviewers. We strongly encourage code deposition in a community repository (e.g. GitHub). See the Nature Portfolio guidelines for submitting code & software for further information.

Data

Policy information about availability of data

All manuscripts must include a data availability statement. This statement should provide the following information, where applicable:

- Accession codes, unique identifiers, or web links for publicly available datasets
- A description of any restrictions on data availability
- For clinical datasets or third party data, please ensure that the statement adheres to our policy

All data for BMFF and BM segmentation volumes have been uploaded to the UKBB (upload ID 5858), where they will be available to any individuals with an approved UKBB project. Researchers can apply for UKBB access via the UKBB Access Management System (https://ams.ukbiobank.ac.uk/ams/). Data used for LDSC GWAS were obtained from GWAS catalog; Pubmed IDs and URLs for the relevant studies from GWAS catalog are presented in Supplementary Data files 32-36, 56-59, and 71-74. For TWAS, pre-computed gene expression weights from GTEx v8 for adipose and skeletal muscle tissues were used as downloaded from the FUSION (http://gusevlab.org/projects/fusion/). The remaining data are reported in the Supplementary Data files

Research involving human participants, their data, or biological material

Policy information about studies with human participants or human data. See also policy information about sex, gender (identity/presentation), and sexual orientation and race, ethnicity and racism.

Reporting on sex and gender	We used biological sex in the study. It was determined based on the participants' genotypes.<br><br>We conducted sex-specific GWAS analyses for BMFF of the first and the second batch for the four bone regions respectively. To investigate sex-specific genetic associations, we further performed sensitivity meta-analyses of white population for the two batches in male and female groups.
Reporting on race, ethnicity, or other socially relevant groupings	We sub-grouped participants based on their ancestry as 'white' and 'Non-white' in the study. It was determined based on genetic ethnic grouping (UK Biobank data field 22006, "Genetic ethnic grouping") [https://biobank.ctsu.ox.ac.uk/crystal/crystal/docs/genotyping_qc.pdf]. The "non-white' participants were further categorized into 'Asian', 'Black' and 'non-white mixed ethnic group' according to UK Biobank data-field 21000 ("Ethnic background"), as self-reported by participants.<br><br>We performed multi-ancestry meta-analyses of white and non-white populations for each bone region using MR-MEGA (v0.20).
Population characteristics	Population characteristics are described in Table 1 and Supplementary Data 3-4, 6, and 37-38.
Recruitment	Recruitment of participants for the UK Biobank is described by Sudlow et al 2015 (doi: 10.1371/journal.pmed.1001779) and Littlejohns et al 2020 (doi: 10.1038/s41467-020-15948-9), which we cite in the main manuscript.
Ethics oversight	UKBB has approval from the North West Multi-Centre Research Ethics Committee (MREC) as a Research Tissue Bank (RTB) approval (Ref: 11/NW/0382). Data for this work were obtained under the approved UKBB project application (ID 48697).

Note that full information on the approval of the study protocol must also be provided in the manuscript.

Field-specific reporting

Please select the one below that is the best fit for your research. If you are not sure, read the appropriate sections before making your selection.

☒ Life sciences ☐ Behavioural & social sciences ☐ Ecological, evolutionary & environmental sciences

For a reference copy of the document with all sections, see nature.com/documents/nr-reporting-summary-flat.pdf

Life sciences study design

All studies must disclose on these points even when the disclosure is negative.

Sample size	Sample sizes were not predetermined but instead resulted from the availability of UK Biobank MRI data and the generation of reliable segmentation outputs from deep learning.
Data exclusions	We describe data exclusions in the Methods, within the subsection named 'Principal component analysis and MRI image error checking to identify technical outliers from deep learning segmentation'. Quality control for GWAS, including reasons for excluding any individuals or genetic variants, is further described in the Methods subsections entitled 'Genome-wide association analyses' and 'GWAS meta-analysis of BMFF'.
Replication	We measured BMFF in two batches (batch 1 and batch 2) based on availability of MRI data released by UK Biobank. These two batches were independently generated, and therefore batch 2 served as a replication cohort for batch 1. GWAS was performed independently in the two batches, which confirmed the reproducibility of the GWAS findings and allowed us to conduct a meta-GWAS of the two batches.
Randomization	This was a genetic association study. Allocation by genotype.
Blinding	This was a genetic association study, ie observational design. So no blinding was used.

Reporting for specific materials, systems and methods

We require information from authors about some types of materials, experimental systems and methods used in many studies. Here, indicate whether each material, system or method listed is relevant to your study. If you are not sure if a list item applies to your research, read the appropriate section before selecting a response.

Materials & experimental systems

n/a	Involved in the study
☒	☐ Antibodies
☒	☐ Eukaryotic cell lines
☒	☐ Palaeontology and archaeology
☒	☐ Animals and other organisms
☐	☒ Clinical data
☒	☐ Dual use research of concern
☒	☐ Plants

Methods

n/a	Involved in the study
☒	☐ ChIP-seq
☒	☐ Flow cytometry
☒	☐ MRI-based neuroimaging

Clinical data

Policy information about clinical studies

All manuscripts should comply with the ICMJE guidelines for publication of clinical research and a completed CONSORT checklist must be included with all submissions.

Clinical trial registration	*Provide the trial registration number from ClinicalTrials.gov or an equivalent agency.*
Study protocol	*Note where the full trial protocol can be accessed OR if not available, explain why.*
Data collection	*Describe the settings and locales of data collection, noting the time periods of recruitment and data collection.*
Outcomes	*Describe how you pre-defined primary and secondary outcome measures and how you assessed these measures.*

Plants

Seed stocks	N/A
Novel plant genotypes	N/A
Authentication	N/A
